# Nanobody-based trispecific T cell engager (Nb-TriTE) enhances therapeutic efficacy by overcoming tumor-mediated immunosuppression

**DOI:** 10.1186/s13045-023-01507-4

**Published:** 2023-11-29

**Authors:** Ziqiang Ding, Shuyang Sun, Xuan Wang, Xiaomei Yang, Wei Shi, Xianing Huang, Shenxia Xie, Fengzhen Mo, Xiaoqiong Hou, Aiqun Liu, Xiaobing Jiang, Zhuoran Tang, Xiaoling Lu

**Affiliations:** 1 School of Basic Medical Sciences/College of Stomatology/Hospital of Stomatology/Guangxi Key Laboratory of Nanobody Research/Guangxi Nanobody Engineering Research Center/Laboratory Animal Center/Pharmaceutical College/Affiliated Tumor Hospital, Guangxi Medical University, Nanning, 530021 China; 2 Department of Neurosurgery, Union Hospital, Tongji Medical College, Huazhong University of Science and Technology, Wuhan, 430022 China

**Keywords:** Nanobody (Nb), Trispecific T cell engager (TriTE), Fibroblast activation protein (FAP), Programmed cell death 1 (PD-1), Solid tumor immunotherapy

## Abstract

**Background:**

T cell engagers (TCEs) have been established as an emerging modality for hematologic malignancies, but solid tumors remain refractory. However, the upregulation of programmed cell death 1 (PD-1) is correlated with T cell dysfunction that confer tumor-mediated immunosuppression. Developing a novel nanobody-based trispecific T cell engager (Nb-TriTE) would be a potential strategy to improve therapeutic efficacy.

**Methods:**

Given the therapeutic potential of nanobodies (Nbs), we first screened Nb targeting fibroblast activation protein (FAP) and successfully generated a Nb-based bispecific T cell engager (Nb-BiTE) targeting FAP. Then, we developed a Nb-TriTE by fusing an anti-PD-1 Nb to the Nb-BiTE. The biological activity and antitumor efficacy of the Nb-TriTE were evaluated in vitro and in both cell line-derived and patient-derived xenograft mouse models.

**Results:**

We had for the first time successfully selected a FAP Nb for the generation of novel Nb-BiTE and Nb-TriTE, which showed good binding ability to their targets. Nb-TriTE not only induced robust tumor antigen-specific killing, potent T cell activation and enhanced T cell function in vitro, but also suppressed tumor growth, improved survival and mediated more T cell infiltration than Nb-BiTE in mouse models of different solid tumors without toxicity.

**Conclusions:**

This novel Nb-TriTE provides a promising and universal platform to overcome tumor-mediated immunosuppression and improve patient outcomes in the future.

**Supplementary Information:**

The online version contains supplementary material available at 10.1186/s13045-023-01507-4.

## Background

Cancer care is being revolutionized by the T cell-based immunotherapy paradigm, and one promising strategy is T cell engager (TCE) technology [1]. Notably, bispecific T cell engagers (BiTEs) are one of the most widely used therapeutic antibody concepts [2, 3]. The first-in-class BiTE, blinatumomab, which target CD3 and CD19, was approved by the FDA for the treatment of B cell malignancies [4]. BiTE has been shown to induce great in patients with hematological malignancies [5]. However, it remains the major hindrance in solid tumors for their heterogeneity and complexity [6, 7]. In addition, various single chain antibody fragments (scFvs)-based TCE formats do not always efficiently fold and can be prone to aggregation, which may impair TCEs function [8, 9]. Moreover, the production of conventional TCEs seem to be challenged due to difficulty in assembling the correct heavy-light chain fragments (Additional file 1: Figure S1A) [9, 10]. With improvements in the field of antibody engineering, nanobodies (Nbs or VHHs), the smallest antigen-binding functional fragments, are emerging as therapeutic agents [11], and the first nanobody drug, caplacizumab, approved by the FDA in 2019 [12]. Nbs are ~ 15 kDa, which allow their rapid distribution and deep diffusion into solid tissues [13, 14]. In addition, Nbs are unaffected by the shuffling of the heavy and light chains and compensate for the shortcomings of scFvs, making it easier to fuse into trispecific or trivalent constructs [8, 15–17]. Not only that, Nbs retain full functionality against their target molecules and bind them with excellent affinity, making them ideal antibody formats for application in the clinic [8, 18]. Accordingly, based on the next generation of potential therapeutic Nb-based agents, our group innovatively proposed a nanobody-based BiTE (Nb-BiTE) platform targeting CD105 (also known as endoglin) in previous research [19], which has been proven to be highly effective in mediating CD105-positive cell lysis in vitro and in mouse xenograft models. However, despite promising results in animal models, BiTEs using either scFvs or Nbs still unable to achieve therapeutic efficacy in solid tumor clinical trials due to the immunosuppressive tumor microenvironment (TME) and, particularly, the dense fibrotic stroma, which can significantly diminish treatment efficacy [6, 20–22]. Therefore, we still need to further augment the efficacy of Nb-BiTE to guide the development of optimal immunotherapy strategies.

Typically, solid tumor growth is accompanied by alterations in the TME and immune inhibitory receptors (IRs) [23, 24]. During immune escape, the presence of immune checkpoint molecules, such as programmed cell death 1 (PD-1) and its ligand programmed death-ligand 1 (PD-L1), would hamper the tumor killing activity of CD8^+^ T cells [25, 26]. This effect is supported by the evidence that targeting immune checkpoint pathways with antibody blockade can partially reverse the depletion of CD8^+^ T cells and T cell function [27–31]. Furthermore, given the expression of multiple checkpoint inhibitory receptors by activated T cells limits the activity of BiTE molecules in solid tumors, and immune checkpoint blockade can reinvigorate durable antitumor responses in multiple cancer types. In particular, it has been reported that blockade of the PD-1/PD-L1 inhibitory pathway can augment the killing capacity of the CD33/CD3 BiTE antibody construct AMG 330 by reversing T cell-induced immune escape mechanism [32, 33]. Thus, effective immune checkpoint blockade enhances the immune response while suppressing immune evasion in cancer cells, providing a rationale for engineering immune checkpoint inhibitor therapy into BiTE molecules.

Herein, we innovatively design a novel nanobody-based trispecific T cell engager (Nb-TriTE) that is composed of two Nbs targeting CD3ε on T cells and tumor associated antigen (TAA) on cancer-associated fibroblasts (CAFs), and the exclusive use of a third Nb targeting immune checkpoint as building blocks of the immunosuppressive signaling pathway. Here, we hypothesize that the Nb-TriTE utilizes three specific Nbs as three anchors that can promote T cell-mediated tumor cytotoxicity by restoring durable immune signaling connections between T cells and CAFs, thereby overcoming immune evasion and eliminating immunosuppression. However, while solid tumor therapy with TCE antibody constructs remains an attractive treatment approach, the selection of appropriate targets is crucial. Dense CAFs exhibit high expression of fibroblast activation protein (FAP) in the immunosuppressive TME of various solid tumors [34–39]. In addition, one of the major checkpoint pathways represents a tumor-induced immunosuppressive milieu mediated by the PD-1/PD-L1 axis of high PD-1 expression on activated T cells interacting with PD-L1 on tumor cells [40]. A more recent phase II trial of simlukafusp alfa (FAP-IL2v) in combination with atezolizumab (anti-PD-L1) for advanced/metastatic squamous cell carcinoma showed great therapeutic potential (NCT03875079).

Overall, in this study, to exemplify the novel Nb-TriTE, we first screened Nbs targeting human FAP from phage display libraries and successfully prepared Nb-BiTE targeting FAP. We then generated an innovative Nb-TriTE by fusing PD-1 Nb [41] that was screened in the previous study to the Nb-BiTE. Correspondingly, we demonstrated that Nb-TriTE could redirect to T cells specificity target FAP^+^ CAFs and additionally overcome immunosuppression by PD-1 immune checkpoint blockade, thereby promoting T cell-mediated cytotoxicity by restoring durable immune synapses and signaling connections between T cells and tumor cells. To our knowledge, this first attempt at constructing the novel Nb-TriTE provides a promising therapeutic platform for enhancing TCE-mediated antitumor effects. This strategy may also enhance the ability of T cells to kill tumor cells. Therefore, the novel Nb-TriTE immunotherapeutic platform may be a universal, TCE technology developed to potentially improve patient outcomes in the clinic.

## Materials and methods

### Cell culture and experimental animals

HepG2, U87, PANC1, and 293T cells were cultured in DMEM supplemented with 10% fetal bovine serum (FBS), penicillin, and streptomycin. Peripheral blood mononuclear cells (PBMCs) were isolated from healthy donors by Ficoll density centrifugation. Recombinant human IL-2 (PeproTech, UK) was added for T cell culture at a concentration of 300 IU/mL. PBMCs and Jurkat cells were cultured in RPMI 1640 supplemented with 10% FBS, penicillin, and streptomycin. Stably transfected 293T-PD-1 and HepG2-FAP cells were cultured in DMEM with 10% FBS, penicillin, and streptomycin and supplemented with puromycin (1 μg/mL). All cells were incubated at 37 °C in an incubator with 5% CO_2_.

Specific pathogen-free (SPF), nonobese diabetic/severe combined immunodeficiency (NOD/SCID) mice (age 4–6 weeks) deficient in both B and T cells were purchased from Vital River (Beijing, China) and maintained in our SPF experimental animal facility. All animal experiments were approved by the Institutional Animal Care and Use Committee (IACUC) of Guangxi Medical University.

### Generation of nanobodies

FAP Nbs were screened through phage display according to our previously described methods [19, 41–43]. FAP Nbs with His-tag were amplified under the induction of isopropyl β-D-1-thiogalactopyranoside (IPTG) (Solarbio, China) under optimal conditions and purified using Ni^2+^–NTA affinity columns. The purified FAP Nbs were analyzed by sodium dodecyl sulfate–polyacrylamide gel electrophoresis (SDS‒PAGE). The binding affinity of FAP Nbs was determined by surface plasmon resonance (SPR) assay, and the specific binding ability was detected by flow cytometry on a FACS Canto analyzer (BD Biosciences, USA).

### Expression, purification and validation of Nb-TriTE

Briefly, genetic engineering technology was used to link the anti-hCD3ε Nb and anti-hPD-1 Nb screened out in previous research [19, 41] with the anti-FAP Nb, synthesized by GenScript Biotechnology Co., Ltd (Nanjing, China) through a (GGGGS)_3_ flexible linker which was cloned into the pET-30a vector (containing His-tag and Flag-tag) and expressed in *E. coli* BL21 (DE3). Bacterial cultures were inoculated into 1 L of terrific broth (TB) with 50 μg/mL kanamycin and grown at 37 °C to an A_600_ value of 2.0. Nb-TriTE expression was induced with 0.5 mM IPTG at 16 °C for 16 h under optimal expression conditions. Then, the cultures were centrifuged at 12,000 rpm for 30 min, and the bacterial pellets were resuspended in phosphate-buffered saline (PBS) buffer, lysed by ultrasonication and stored at − 80 °C. Inclusion bodies were prepared by extensively washing bacterial material with PBS. After denaturation in 8 M urea, the material was subjected to gradient dialysis and then was sterile filtered through a 0.22-μm filter. The Nb-TriTE was purified by Ni^2+^–NTA affinity resin (GenScript, China) according to the manufacturer’s instructions. Protein purity was subjected to SEC analysis and SDS‒PAGE analysis under reducing conditions and visualized with Coomassie blue staining.

### Western blotting

Nb-TriTE expression was detected by Western blotting with horseradish peroxidase (HRP)-conjugated anti-His-tag antibody (Abcam, USA). For Western blotting analysis, purified proteins were analyzed by SDS‒PAGE under reducing conditions and transferred onto polyvinylidene fluoride (PVDF) membranes using wet/tank blotting systems (Bio-Rad, USA). After incubation with 5% skim milk blocking solution, proteins were detected with HRP-conjugated anti-His-tag antibody diluted 1:3000. The blots were visualized with an enhanced chemiluminescent reagent (BeyoECL Plus Lit, Beyotime, China) and analyzed using Image Lab software (Bio-Rad, USA).

### Enzyme-linked immunosorbent assay (ELISA)

ELISA was performed using commercially available kits (Liankebio, China) to measure IL-2, IFN-γ, TNF-α, and GzmB and PRF1 (Elabscience, China) levels following the manufacturers’ instructions. In brief, serum and cell supernatant samples (100 μL/well) were collected, and diluted antibody solutions were added for 2 h at 37 °C. After washing with PBS with 0.05% Tween 20 (PBST) buffer and incubation with HRP-conjugated avidin for 45 min, the OD_450_ value was measured after incubation with tetramethylbenzidine (TMB) for 10 min using a microplate reader (Tecan, Mannedorf, Switzerland). The cytokine levels in the samples were calculated based on the established standard curves.

The PD-1/PD-L1 blocking activities of Nb-TriTE were measured using an ELISA assay with hPD-1 (ACRO, PD1-H5257) as the capture reagent and biotinylated hPD-L1 (ACRO, PD1-H82E5) as the detection reagent. In addition, Nb-TriTE simultaneous binding ability to antigens was assessed by a two-step sandwich ELISA with hFAP, hCD3 epsilon, and hPD-1 (ACRO, FAP-H5263, CDE-H5256, PD1-H5257) as the capture reagents, biotinylated hFAP, biotinylated hCD3 epsilon, and biotinylated hPD-1 (ACRO, FAP-H82Q6, CDE-H82E1, PD1-H82E4) as the detection reagents, the OD_450_ value was measured using a microplate reader (Tecan, Mannedorf, Switzerland).

### Flow cytometry

Incubation with a PE-conjugated anti-human FAP antibody (R&D Systems, USA) was performed to determine the expression of target cells, an APC-conjugated anti-human PD-L1 antibody (Biolegend, USA) was performed to determine the expression of target cells. Nb-TriTE (2 μg/mL) was used in conjunction with a PE-conjugated anti-His-tag antibody (R&D Systems, USA) and to analyze binding ability. Flow cytometry analysis was performed on a FACS Calibur flow cytometer (BD Biosciences, USA), and data were evaluated using FlowJo software.

### T cell activation assay

To determine in vitro T cell activation, phenotyping and proliferation assays were performed. Enriched T cells were incubated with target cell lines at a final effector-to-target ratio of 10:1 in the presence of Nb-TriTE and other control molecules. The supernatant was removed after incubation for 24 h at 37 °C, and T cell activation was assessed by determining CD25, CD69 and CD107a expression by a PE-conjugated anti-CD69 antibody, a PE-conjugated anti-CD25 antibody and an APC-conjugated anti-CD107a antibody (Biolegend, USA). After incubation for 14 days at 37 °C, a PE-Cy7-conjugated anti-CD45RA antibody and an APC-conjugated anti-CD62L antibody (Biolegend, USA) were added and incubated for 30 min at 4 °C to determine the proportion of memory T cells. Samples were acquired on a FACS Calibur flow cytometer (BD Biosciences, USA), and data were processed using FlowJo software.

### Proliferation assay

T cell proliferation was evaluated using a PKH26 assay kit according to the manufacturer’s protocol (Sigma, USA). Briefly, 1 × 10^5^ mitomycin C-treated tumor cells were incubated with PKH26-labeled PBMCs (E:T = 10:1) in the presence of either Nb-TriTE or control molecules for an additional 5 days. T cell proliferation was assessed by flow cytometry, and quantified as the % loss of PKH26 in each generation.

### Cytotoxicity assay

The ability of Nb-TriTE to redirect cytotoxicity was assessed using a flow cytometry-based cytotoxicity assay. In brief, target cell lines were labeled with a CFSE (5,6-carboxyfluorescein diacetate, succinimidyl ester) fluorescent cell labeling kit following the manufacturer's protocol (Invitrogen, USA) and then incubated with effector T cells at an effector to target cell (E:T) ratio of 1:1, 5:1 or 10:1 and increasing concentrations of Nb-TriTE or control molecules. The cell mixture was incubated for 16 h at 37 °C and 5% CO_2_, and then stained with a propidium iodide (PI) (Sigma-Aldrich, USA) according to the manufacturer's protocol, and the percentage of CFSE^+^PI^+^ cells in each group was determined by flow cytometry as the percentage of specific lysis. Specific lysis (%) = (fluorescence (sample lysis) − fluorescence (spontaneous lysis))/(fluorescence (maximum lysis) − fluorescence (spontaneous lysis) × 100.

### In vivo efficiency study

HepG2-FAP cells (1 × 10^6^ cells/mouse), and PANC1 cells (1 × 10^6^ cells/mouse) mixed with CAFs at a ratio of 2:1 in PBS were implanted by subcutaneous inoculation into the lower dorsal flank or axilla of NOD/SCID mice to establish a cell-derived xenograft (CDX) model. Seven days after implantation, the mice were randomized into different groups (*n* = 5) and treated with PBMCs (1 × 10^7^ cells/mouse) in 100 µL of PBS intravenously via tail vein injection. The mice were intravenously injected with Nb-TriTE (20 µg/mouse) or control molecules every day for a total of 6 days, and an equal volume of PBS was used in the control group. Tumor growth was monitored every 4 days, tumor volume was calculated with calipers using the formula (length × width^2^)/2, and the body weight of the mice was also monitored. Mice were euthanized when they displayed obvious weight loss, tumor ulcerations, or a tumor size larger than 2000 mm^3^.

To establish the intracranial orthotopic CDX model, the anesthetized NOD/SCID mice were fixed on a small animal stereotaxic instrument with a mouse adapter, and 1 × 10^5^ U87 cells resuspended in 5 μL PBS were injected to the right of the sagittal suture and 2 mm from the posterior of the anterior fontanelle. Seven days after successful engraftment, 1 × 10^6^ PBMCs resuspended in 100 μL PBS were transplanted and followed by daily intravenous injections of Nb-TriTE or Nb-BiTE (20 µg) for a total of 6 days. The treated mouse brains that contained tumors were harvested for histological examination.

### Patient-derived tumor xenograft model

The establishment of the patient-derived xenograft (PDX) model was performed as previously described [42, 44, 45]. In brief, human liver cancer cells (P0) were dissociated in suspension buffer, and fragments forming the same tumor mass were mixed with Matrigel matrix (Corning, USA) and subcutaneously inoculated into the lower dorsal flank or axilla of NOD/SCID mice (P1). Seven days after successful engraftment, PBMCs were transplanted and followed by daily intravenous injections of 20 µg Nb-TriTE or Nb-BiTE for a total of 6 days. Tumor length and width were measured every 4 days with a caliper, and tumor volumes were calculated by using the formula above. For the short-term study, ex vivo analysis was performed on harvested tumors, spleens and blood, and part of the tumor was fixed with formalin and embedded in paraffin for histological evaluation or immunohistochemistry.

### RNA-seq assay

The RNA-seq assay was performed by Gene Denovo Biotechnology Co. Ltd. (Guangzhou, China). Total RNA was extracted from the tumor tissues using the TRIzol reagent kit (Invitrogen, USA) according to the manufacturer’s protocol. The ratio of absorbance at 260 nm and 280 nm (A260/A280) was used to assess RNA quality, and RNA integrity was determined by 1.5% agarose gel electrophoresis. Eukaryotic mRNA was then enriched using oligo(dT) beads, and the enriched RNA was used for RNA sequencing library preparation using the NEBNext Ultra RNA Library Prep Kit (New England Biolabs, #E7530, USA). The purified double-stranded cDNA fragments were end-repaired and ligated to Illumina sequencing adapters. The ligation reaction was purified using AMPure XP Beads (1.0), and the cDNA was amplified by polymerase chain reaction (PCR). The resulting cDNA library was sequenced using NovaSeq 6000 (Illumina). Reads mapping was then scaled to transcripts per million (TPM). Differentially expressed genes (DEGs) were identified when the gene showed a > two fold change and adjusted *p* < 0.05. The DEG analysis results were visualized by a heatmap. Immune signatures were identified with analyses that use public gene lists.

### Histological studies

For the terminal deoxynucleotidyl transferase dUTP nick-end labeling (TUNEL) assay, samples were analyzed using a one-step TUNEL detection kit (Beyotime, China) according to the instructions. Moreover, for immunohistochemistry (IHC) staining of Ki67 expression, paraffin-embedded sections were processed by microwave heating for antigen retrieval, and nonspecific endogenous peroxidase activity was blocked by incubation with 3% hydrogen peroxide and goat serum. Ready-to-use anti-Ki67 antibody (MX006, MXB, China) was added and incubated. Afterward, the slides were incubated with the corresponding secondary antibody according to standard protocols. Expression was visualized by diaminobenzidine (DAB) staining detection. The samples were imaged using a Nikon upright microscope.

To assess the in vivo toxicity of the Nb-TriTE, mice received intravenous injections of PBS and the Nb-TriTE. After treatment, the liver, heart, spleen, lung and kidney were isolated from sacrificed mice and were subjected to routine formalin fixation and paraffin embedding. Representative 5-μm sections were stained with hematoxylin–eosin (HE) according to standard protocols. Mouse serum IL-6 and IL-1β levels were measured using ELISA kits (Liankebio, China) according to the manufacturers’ instructions. Levels of alanine aminotransferase (ALT) and aspartate transaminase (AST) were measured using an automatic biochemical analyzer (Catalyst One, IDEXX, USA).

### Statistical analysis

Statistical analysis was conducted using GraphPad Prism 9.0 software (GraphPad, USA) applying one-way analysis of variance (ANOVA) with Tukey’s multiple comparison or Student's *t* test. *P* < 0.05 was considered statistically significant (*P < 0.05; **P < 0.01; ***P < 0.001; ****P < 0.0001). Data represent the mean ± standard deviation (SD).

## Results

### Generation and verification of FAP Nbs

**Fig. 1:**
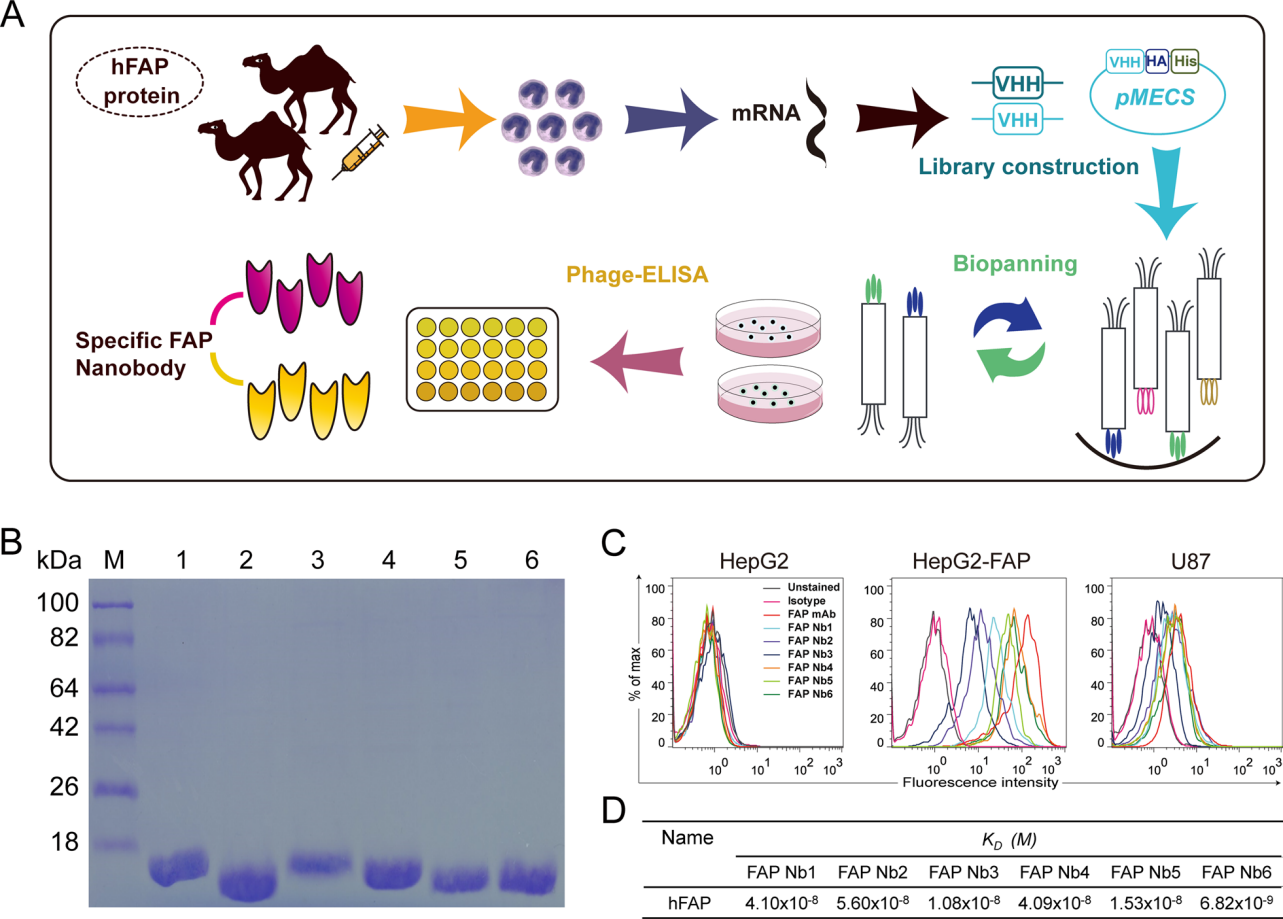
Construction of the Nb library and screening for hFAP-specific Nbs. **A** Schematic flow of the general procedure for the preparation of hFAP-specific Nbs. **B** SDS‒PAGE gel analysis for six hFAP Nbs. **C** Flow cytometry analysis of hFAP Nbs that strongly bind to FAP^+^ HepG2-FAP cells and U87 cells but not to FAP^−^ HepG2 cells. **D** The binding affinity index KD (M) value of hFAP Nbs was measured by SPR analysis

Briefly, purified hFAP Nbs were obtained following a previously reported procedure [19, 41–43], and a schematic diagram is shown (Fig. 1A). First, we obtained the first-step PCR products of the VHH-CH2 fragment (a target band of ~ 700 bp) (Additional file 1: Figure S2A), which was then used as a template for the second-step PCR, from which we obtained a ~ 400 bp VHH fragment (Additional file 1: Figure S2B). An immune Nb phage display library was constructed after endonucleases digestion. Then, the VHH genes were cloned into pMECS plasmids and then electrotransferred into *E. coli* TG1 cells, and the FAP Nb library capacity was 2.25 × 10^8^ CFU/mL (Additional file 1: Figure S2C), which was calculated by counting the number of colonies in gradient dilution plates. The correct insertion rate was estimated by 24 randomly selected after PCR analysis (Additional file 1: Figure S2D). After three rounds of panning, the positive colonies were enriched in FAP-specific Nbs compared to the control colonies (+/−) (Additional file 1: Figure S2E). Ninety-six randomly selected colonies were screened by phage ELISA, and 24 clones were selected as positive (ratio higher than 3) (Additional file 1: Figure S2F). Subsequently, we selected six different amino acid sequences of specific Nbs, and the purified hFAP Nbs showed a target band of ~ 15 kDa by SDS‒PAGE (Fig. 1B). Flow cytometry analysis demonstrated that all selected FAP Nbs bound specifically to FAP-positive target cells (HepG2-FAP and U87), while they did not bind to FAP-negative target cells (HepG2) (Fig. 1C). The SPR analysis results showed that FAP Nbs have good affinity with dissociation constants (KD) of 10^−8^–10^−9^ M (Fig. 1D). Therefore, our results showed that the FAP-specific Nbs immune library was successfully constructed, and FAP Nb4 was selected for further research due to its high yield of expression and good affinity.

### Design, generation and identification of the Nb-TriTE and Nb-BiTE

**Fig. 2:**
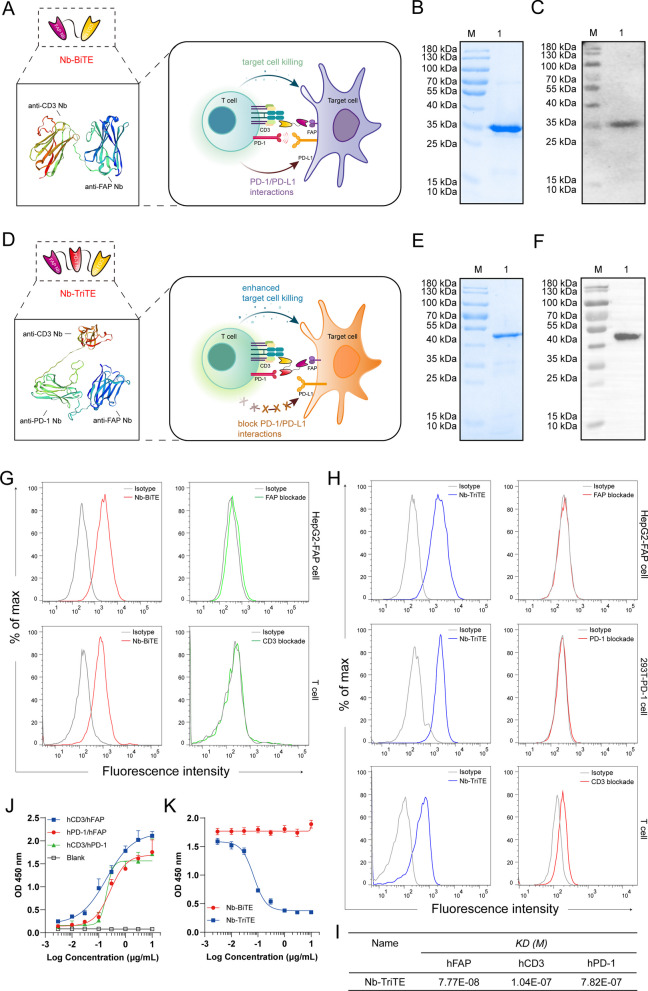
Generation of Nb-TriTE and Nb-BiTE. Schematic drawing of a Nb-BiTE molecule (**A**), a Nb-TriTE molecule (**D**) and the protein model for them was proposed by using the SWISS-MODEL website tool. (www.ExPASy.org/resources/swiss-model). The Nb-BiTE molecule consists of a FAP Nb that targets CAFs and a CD3ε Nb that engages T cells. The Nb-TriTE molecule was based on the Nb-BiTE molecule with the addition of a PD-1 Nb that blocks the PD-1/PD-L1 axis. **B** SDS‒PAGE analysis of purified Nb-BiTE protein was visualized by Coomassie blue staining. Lanes: M- molecular weight marker, 1- Nb-BiTE protein. **C** Western blot identification of purified Nb-BiTE protein was probed with anti-His-tag antibody, M- molecular weight marker; 1- Nb-BiTE protein. **E** SDS‒PAGE analysis of purified Nb-TriTE protein was visualized by Coomassie blue staining. Lanes: M- molecular weight marker, 1- Nb-TriTE protein. **F** Western blot identification of purified Nb-TriTE protein was probed with anti-His-tag antibody, M- molecular weight marker; 1-Nb-TriTE protein. **G** Binding analysis of Nb-BiTE to FAP-expressing HepG2-FAP cells and CD3-expressing human T cells at a concentration of 2 μg/mL measured by flow cytometry. **H** Binding analysis of Nb-TriTE to FAP-expressing HepG2-FAP cells, PD-1-expressing 293T-PD-1 cells and CD3-expressing human T cells at a concentration of 2 μg/mL measured by flow cytometry. Blocking assay using recombinant target antigens that substantially interfere with binding ability, representative histograms for each group. **I** The binding affinity of the Nb-TriTE was determined by SPR analysis. **J** Nb-TriTE simultaneous binding ability to two target antigens (hCD3ε/hFAP, hPD-1/hFAP and hCD3ε/hPD-1) was assessed by a two-step sandwich ELISA assay. **K** The effect of Nb-TriTE on blocking PD-1/PD-L1 interaction was assessed by ELISA. All data represent the mean ± standard deviation from 3 independent experiments

To specifically redirect T cells to FAP^+^ tumor cells supplied with a T cell-restricted PD-1/PD-L1 blockade, we developed a Nb-TriTE antibody by fusing an anti-PD-1 Nb to a Nb-BiTE molecule targeting FAP with a flexible (Gly_4_Ser)_3_ linker. A nontargeting irrelevant Nb-BiTE/TriTE (Irrelevant ctr) was used as a control group. Protein homology modeling of the Nb-TriTE and Nb-BiTE was performed using the SWISS model (https://swissmodel.ExPASy.org) (Fig. 2A, D). In terms of molecular formats, the Nb-TriTE format differs from conventional TriAbs [46] and recently modified TriTEs [47], and the same is true for the Nb-BiTE format (Additional file 1: Figure S1A). A construct of Nb-TriTE was expressed in transformed *E. coli *BL21 (DE3), and Coomassie-stained SDS‒PAGE showed that 0.5 mM IPTG, 16 °C, and 16 h were the best expression conditions. The Nb-TriTE was then isolated from insoluble inclusion bodies and purified by a Ni^2+^–NTA column (Additional file 1: Figure S3A–D). Coomassie-stained SDS‒PAGE and SEC analysis showed the purified Nb-BiTE and Nb-TriTE proteins with single bands (> 90% purity) (Fig. 2B, E, Additional file 1: Figure S3E–F). Furthermore, the identity of the purified proteins was verified by Western blot analysis, which confirmed a specific protein band consistent with the molecular weights with predicted molecular weights of ~ 35 kDa and ∼ 50 kDa, respectively (Fig. 2C,F). To determine the optimal configurations of PD-1 Nb and CD3ε Nb in alternative positions of the Nb-TriTE, we evaluated them by cytokine release assays in vitro. T cell stimulation was determined based on the secretion levels of IL-2 and IFN-γ (Additional file 1: Figure S4A–B) in the supernatant after 24 h measured by ELISA. The results showed that proximal-PD-1 Nb × distal- CD3ε Nb was identified as the optimal positioning, and thus, this configuration was then selected for pairing with a FAP Nb in the Nb-TriTE format.

### Binding properties of the Nb-TriTE and Nb-BiTE

Binding assays were performed to examine the binding of the Nb-TriTE and Nb-BiTE to various target cells by flow cytometry and SPR assay, respectively. Our results demonstrated that the Nb-BiTE can specifically bind to HepG2-FAP and T cells but not to HepG2 cells (Fig. 2G, Additional file 1: Figure S5A-B and S5I). Moreover, the results also showed that the Nb-TriTE specifically bound to HepG2-FAP and 293T-PD-1 cells but not to parental HepG2 or 293T cells (Fig. 2H, Additional file 1: Figure S5C-D and S5J). In addition, the Nb-TriTE was also found to specifically bind to CD3-expressing T cells and Jurkat cells (Fig. 2H, Additional file 1: Figure S5G-H). Importantly, we performed a blocking assay with associated recombinant proteins that interfered with Nb-TriTE and Nb-BiTE binding. We observed that the PD-1 recombinant protein was able to block the binding of Nb-TriTE to 293T-PD-1 cells, and similar results were observed when the FAP or CD3ε recombinant proteins were used in a blocking assay (Fig. 2G, H**, **Additional file 1: Figure S5A–D and S5G–H). Interestingly, CD3ε blockade was not as efficient compared to PD-1 blockade or CD3ε + PD-1 co-blockade, in which the Nb-TriTE was able to interact with PD-1 molecules on T cells (Additional file 1: Figure S5E, F). Furthermore, we measured the affinity constants of the Nb-TriTE against recombinant hFAP, hPD-1 and hCD3ε antigens by SPR assay, indicating that the KD (M) for Nb-TriTE binding to individual antigens was 10^−7^ ~ 10^−8^ M (Fig. 2I). Notably, results showed that Nb-TriTE can simultaneously bind to two target antigens that do not preclude binding of another due to steric hindrance effects (Fig. 2J). In addition, Nb-TriTE can block PD-1/PD-L1 interaction in a dose-dependent manner compared with Nb-BiTE (Fig. 2K). Collectively, these results demonstrated the important biological property of binding specificity of the Nb-TriTE and Nb-BiTE.

### Nb-TriTE-mediated T cell activation and proliferation and cytokine secretion

**Fig. 3:**
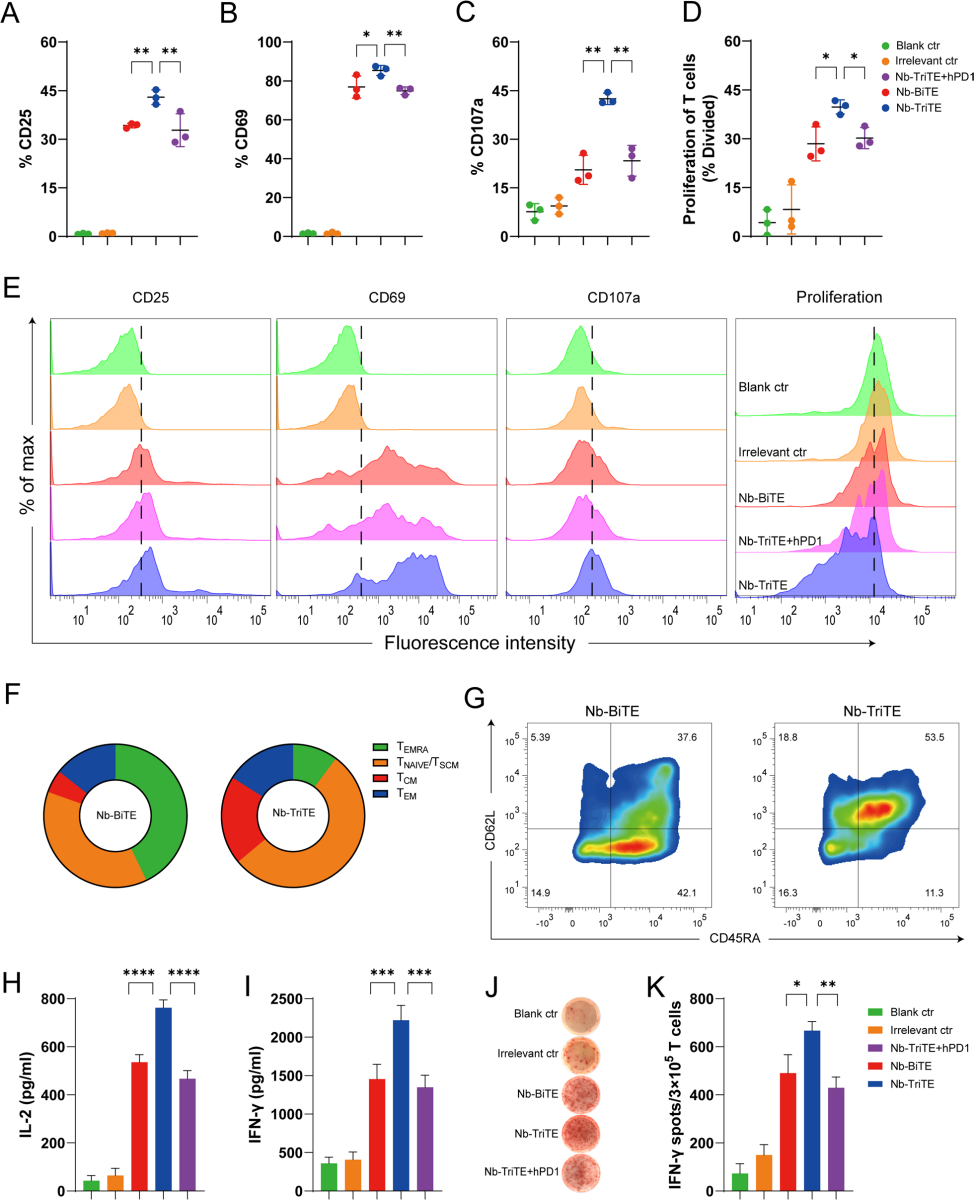
Nb-TriTE enhances T cell activation and function compared with Nb-BiTE. Nb-TriTE-induced T cells upregulation of CD25 (**A**), CD69 (**B**) and CD107a (**C**) in comparison with controls as analyzed by flow cytometry in the presence of HepG2-FAP cells at a 2 μg/mL protein concentration after 24 h and an E:T ratio of 10:1. **D** Proliferation of T cells in response to Nb-TriTE in comparison with other groups was measured by flow cytometry in the presence of HepG2-FAP cells after 5 days and an E:T ratio of 10:1, shown as a percentage divided. **E** Representative histograms of CD25, CD69, and CD107a expression and proliferation indices are shown for each group. **F**–**G** Subpopulation analysis based on CD62L and CD45RA expression induced by the Nb-TriTE or Nb-BiTE after 14 days. CD45RA^−^CD62L^−^cells represent effector memory T (TEM) cells, CD45RA^−^CD62L^+^cells represent central memory T (TCM) cells, and CD45RA^+^CD62L^+^cells represent naive T cells. Pie chart for CD62L/D45RA expression (left). (H-I) Levels of IL-2 and IFN-γ secretion in the presence of HepG2-FAP cells upon addition of Nb-TriTE, Nb-BiTE or Irrelevant ctr and Nb-TriTE + hPD-1 at equimolar concentrations. (J-K) An ELISPOT assay was performed to measure the IFN-γ response of specific T cells for each group. All data represent the mean ± standard deviation from 3 independent experiments

We next investigated the effects of the Nb-TriTE and Nb-BiTE on T cell activation and proliferation and cytokine production. Nb-TriTE- and Nb-BiTE-mediated T cell activation depends on the formation of immunological interaction between T cells and target cells expressing target antigens, and we used the activation markers CD25 and CD69 to assess their ability to activate T cells in vitro. As displayed in Fig. 3, we incubated T cells with the Nb-TriTE and Nb-BiTE in the presence of target HepG2-FAP cells. Strikingly, compared to Nb-BiTE and Nb-TriTE + hPD-1, Nb-TriTE-induced T cells exhibited upregulated surface expression of CD25 and CD69 (Fig. 3A, B). Indeed, the irrelevant control group did not exhibit significant T cell activation. Moreover, the expression level of the degranulation indicator CD107a was higher in the Nb-TriTE-induced group than in the other groups (Fig. 3C). These results confirmed that as expected for Nb-TriTE engagement, the activation signal was prominent, which consequently generated a proper T cell response. Another important hallmark of activated T cells is their proliferative capacity. The ability of the Nb-TriTE to induce the specific proliferation of T cells was evaluated by flow cytometry after 5 days of coculture. T cells underwent multiple rounds of proliferation only when cocultured with Nb-TriTE and mitomycin C-treated, nonproliferating tumor cells together. In FAP^+^ tumor cells, compared to Nb-BiTE and Nb-TriTE + hPD-1 group, Nb-TriTE group led to significant cell divisions (Fig. 3D). Indeed, T cells cocultured with Irrelevant ctr and the blank control (Blank ctr), showed no T cell proliferation. Representative raw flow cytometry data of CD25, CD69, CD107a and proliferation between groups are shown in Fig. 3E. These analyses indicated that Nb-TriTE can significantly induce T cell-specific activation upon target cell stimulation. We further determined the expression of CD62L and CD45RA to define Nb-TriTE-or Nb-BiTE-induced T cell memory subsets and central memory T cells (TCMs, CD45RA^−^ /CD62L^+^), which usually have a higher proliferative potential than naive cells, preferentially reside in lymphoid organs, and readily differentiate into effector cells in response to antigens. The results showed that compared to the Nb-BiTE, the Nb-TriTE increased the proportion of TCMs (Fig. 3F, G), suggesting that Nb-TriTE may enhance proliferation and induce a more persistent antitumor response.

Proinflammatory cytokines are mainly secreted by activated T cells and are involved in T cell cytotoxicity for tumor suppression. We also measured the secretion of IL-2, IFN-γ, GzmB and PRF1 after incubation with target cells upon T cell activation by Nb-TriTE and Nb-BiTE. As shown in Fig. 3H, I, Nb-TriTE induced higher secretion levels of IL-2 and IFN-γ than Nb-BiTE, Nb-TriTE + hPD-1 or control groups in the presence of FAP^+^ target cells. However, Nb-BiTE induced similar cytokine levels to those induced by Nb-TriTE + hPD-1, whereas the equimolar addition of control groups results in lower IL-2 and IFN-γ levels. Similar results were obtained by measuring IFN-γ production in ELISPOT assays (Fig. 3J, K). We also tested the levels of proinflammatory cytokines by ELISA and ELISPOT IFN-γ production after incubation with FAP^−^ HepG2 tumor cells, and no significant changes in IL-2 or IFN-γ secretion (Additional file 1: Figure S6A, B) or the IFN-γ response (Additional file 1: Figure S6C, D) were observed. In addition, compared to Nb-BiTE or control group, Nb-TriTE induced higher GzmB and PRF1 secretion levels after incubation with FAP^+^ tumor cells, but no significant changes in FAP^−^ HepG2 tumor cells (Additional file 1: Figure S6E, F). Therefore, these findings demonstrated that our Nb-TriTE is directly more effective in mediating T cell activation and proliferation. We hypothesize that this effect is likely due to PD-1/PD-L1 checkpoint blockade by the Nb-TriTE and that antigen-specificity is a key factor mediating this activity.

### Nb-TriTE-mediated cytotoxicity to FAP-positive tumor cells in vitro

**Fig. 4:**
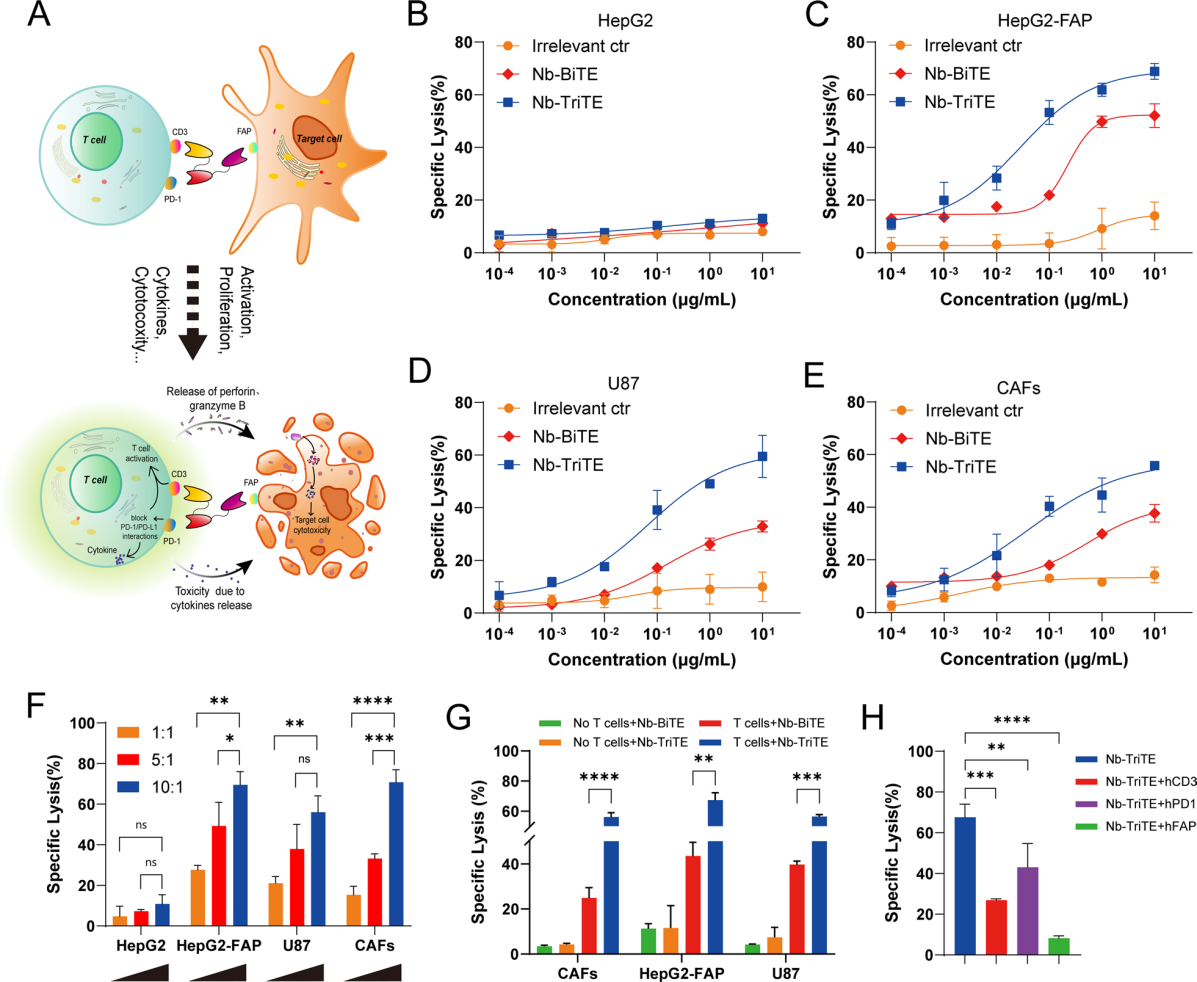
Nb-TriTE enhances the specific lysis of FAP-expressing target cells by T cells in vitro*.* **A** Schematic diagram of enhanced tumor-specific cytotoxic T cells induced by Nb-TriTE due to PD-1 blockade. **B**–**E** Dose-dependent lysis of HepG2 versus HepG2-FAP cells, U87 cells and primary CAF cells by unstimulated T cells varies proportionally with Nb-TriTE concentration (E:T ratio 10:1; incubation time, 16 h). **F** Nb-TriTE-induced cytotoxicity assays of target HepG2, HepG2-FAP, U87 and CAF cells were carried out at E:T ratios of 1:1, 5:1, and 10:1 for 16 h. **G** Cytotoxicity assays of target HepG2-FAP, U87 and CAF cells in the presence of Nb-BiTE and Nb-TriTE or Nb-BiTE (no T cells) and Nb-TriTE (no T cells) were carried out at E:T ratios of 10:1 for 16 h. **H** Cytotoxic lysis of HepG2-FAP in the presence of Nb-TriTE and T cells at a concentration of 2 μg/mL at an E:T ratio of 10:1 after 16 h, which is significantly interfered in the presence of hCD3ε, hPD-1 and hFAP blockade, respectively

We investigated the ability of Nb-TriTE to induce antigen-specific cytotoxicity in vitro and postulated that the increased cytotoxicity of Nb-TriTE leads to an enhanced antitumor response through PD-1/PD-L1 checkpoint blockade (Fig. 4A). Firstly, tumor cells and tumor tissues were tested for FAP and PD-L1 expression by flow cytometry and public datasets showed that their variable expression levels (Additional file 1: Figure S10A–F). After T cells were cocultured with target cells at an E:T ratio of 10:1 in the presence of different concentrations of purified Nb-TriTE, Nb-BiTE or Irrelevant ctr, results showed that the Nb-TriTE efficiently induced T cell cytotoxicity against HepG2-FAP target cells with EC50 values of 0.63 nM compared to the Nb-BiTE (EC50 7.5 nM) (Fig. 4C), but it was inactive against the FAP^−^ cell line HepG2, which was strictly antigen-specific and in a dose-dependent manner (Fig. 4B). Similarly, the Nb-TriTE also redirected the cytotoxicity of T cells toward other human FAP-expressing tumor cells compared with Nb-BiTE, including U87 cells (EC50 1.5 nM vs. 5.19 nM) (Fig. 4D) and primary CAFs (EC50 0.85 nM vs. 17.48 nM) (Fig. 4E). Consistent with the previous characterization, compared to the Nb-BiTE, the Nb-TriTE significantly enhanced target cell lysis. However, no obvious target cell lysis was observed upon incubation with an Irrelevant ctr.

Our results also showed the E:T ratio-dependent FAP^+^ cell killing activity of the Nb-TriTE, which significantly induced the lysis of FAP^+^ target lines (HepG2-FAP, U87 and CAFs). In contrast, no enhanced killing effects on FAP^−^ target HepG2 cells were observed, which demonstrated that the Nb-TriTE-induced T cell killing effect was target cell-specific and E:T ratio-dependent (Fig. 4F). As expected, none of the molecules induced cytotoxic effects without T cells, whereas the Nb-TriTE exhibited a significantly better killing performance in the presence of T cells compared to Nb-BiTE, underscoring that the killing was indeed Nb-TriTE-induced T cells dependent (Fig. 4G). To further investigate whether the possibility of Nb-TriTE either a “cis”-cross-linking event on the T cell surface or a “trans”- interaction between the T cell and the target cell, we generated additional control reagents (Nb-TriTE + hFAP, Nb-TriTE + hPD-1 and Nb-TriTE + hCD3), these data indicate that Nb-TriTE has better in vitro activity for enhancing T cell activity in a trans cell-dependent manner (Fig. 4H), Taken together, we conclude that Nb-TriTE-mediated cytotoxicity is against FAP^+^ target cells and efficiently counteract PD-1-mediated resistance mechanisms and induce the specific lysis of target cells through the synergy of PD-1/PD-L1 checkpoint blockade.

### In vivo antitumor efficacy of the Nb-TriTE in mouse xenograft models

**Fig. 5:**
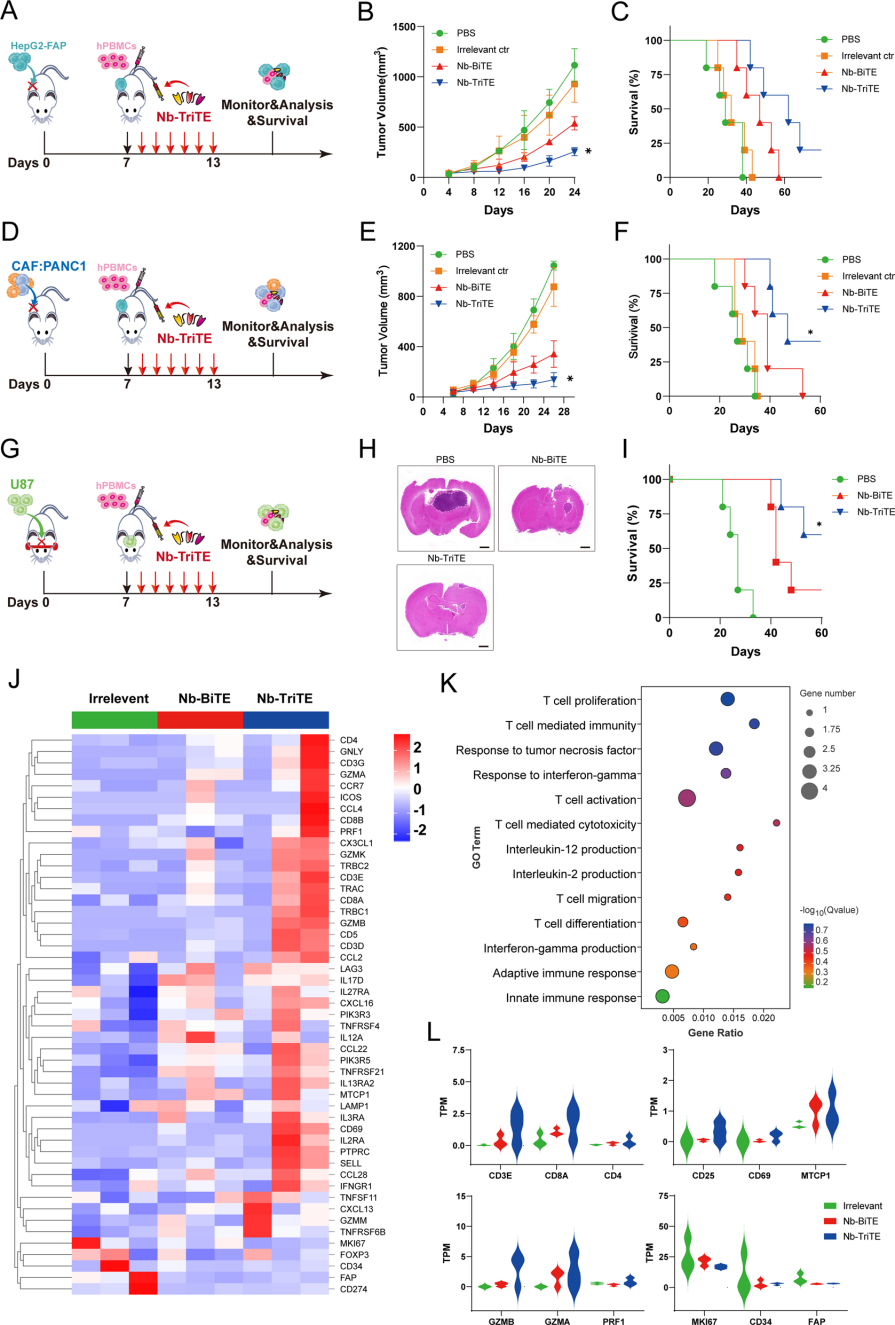
Nb-TriTE treatment enhances antitumor efficacy in multiple cancer types*.* **A** Schematic representation of the experimental timeline. NOD/SCID mice (n = 5 per group) were subcutaneously implanted with 1 × 10^6^ HepG2-FAP tumor cells on day 0, intravenously injected with human PBMCs at a ratio of 1:10 on day 7, and then treated with the Nb-TriTE or the indicated antibodies (20 μg) by daily intravenous infusion for a total of 6 doses. **B** Tumor volume growth curves. **C** Kaplan‒Meier survival curves. **D** Schematic experimental design of CAFs: PANC1 tumor xenograft studies. **E** Tumor volumes, **F** Survival curves of CAFs: PANC1 tumors in NOD/SCID mice treated with the indicated antibodies by daily intravenous infusion (n = 5 per group) **G** Operational diagram of the animal experiment, **H** HE staining of brain, **I** Survival curves of U87 tumors in NOD/SCID mice treated with the indicated antibodies by daily intravenous infusion. **J** Heatmap showed the expression levels of candidate genes in tumor tissues from Irrelevant ctr-treated mice, Nb-BiTE-treated mice or Nb-TriTE-treated mice. **K** GO enrichment analysis of genes in tumor tissues from Nb-BiTE-treated versus Nb-TriTE-treated mice. **L** TPM was used for relative assessment of gene expression levels, such as T cell activation, proliferation and T cell cytotoxicity-associated genes (*n* = 3 independent biological samples)

We then examined the therapeutic efficacy of the Nb-TriTE in vivo. We first established CDX models with cancer types that express FAP: HepG2-FAP cells and a mixture of CAFs and PANC1 cells. On day 0, 1 × 10^6^ tumor cells in 100 μL PBS were subcutaneously injected per tumor, and tumor-bearing mice were randomized into treatment groups and administered via tail vein injection with 1 × 10^7^ expanded T cells derived from PBMCs on day 7. After administration, mice were intravenously injected with the Nb-TriTE, Nb-BiTE, Irrelevant ctr, or PBS daily for 6 days (Fig. 5 A, D). As shown in Fig. 5B, E, the data showed that the tumor growth curves of the PBS and Irrelevant ctr groups were steeper than those of the Nb-TriTE and Nb-BiTE groups, which indicated the highest efficacy of Nb-TriTE in inhibiting tumor growth, and this treatment also resulted in significant improvements in overall survival rates (Fig. 5 C, F). Moreover, the orthotopic glioma CDX model was established by the intracranial implantation of U87 cells (Fig. 5G), and the Nb-TriTE-treated mice had smaller intracranial tumor sizes and improved survival rate (Fig. 5H, I) compare to those in the Nb-BiTE and PBS group, providing evidence for the therapeutic benefit of the Nb-TriTE in preclinical models. No significant differences in body weight were observed with any of the treatments in the three CDX models (Additional file 1: Figure S7A–C). Line graph depicting tumor volume in HepG2-FAP and CAFs: PANC1 tumor-bearing mice (Additional file 1: Figure S7E, F). Therefore, the Nb-TriTE is effective in inhibiting in vivo tumor growth and improving survival in mice. We further confirmed that Nb-TriTE has greater tissue penetration than conventional TriAbs, longer survival time and IHC staining analysis showed that Nb-TriTE is indeed more deeply distributed within tumor tissue compared with conventional TriAbs (Additional file 1: Figure S8A–C).

To further assess the ability of the Nb-TriTE to promote immune cell efficacy, RNA-seq from on the CAFs: PANC1 tumor tissues from the Nb-TriTE treatment group were performed to map the genomic landscape (Fig. 5J), and DEGs associated with the Nb-TriTE group were identified. Moreover, Gene Ontology (GO) enrichment analysis comparing the DEGs between the Nb-BiTE and Nb-TriTE treatment groups revealed that the DEGs associated with Nb-TriTE group were enriched in T cell proliferation and activation, T cell mediated immunity and cytotoxicity, and immune response-associated proinflammatory cytokines TNF, IFN-γ and IL-2 (Fig. 5K). GO enrichment analysis of the DEGs between the Nb-TriTE and control group, the Nb-BiTE and control group (Additional file 1: Figure S9A, B) were also depicted. Furthermore, gene signature analysis revealed that T cell activation, proliferation and cytotoxicity-related key genes in the Nb-TriTE group were upregulated compared with those in the Nb-BiTE and control group (Fig. 5L). For example, T cell types (CD3E, CD8A, CD4), T cell activation genes (CD25, CD69) and proliferation genes (MTCP1), and T cell cytotoxic effector molecules (GzmB, GzmA, PRF1). However, tumor-related genes (MKI67, CD34) and CAFs (FAP) were downregulated in the Nb-TriTE group compared with the other groups. Therefore, the Nb-TriTE is effective in inhibiting in vivo tumor growth and improving survival in mice by enhancing T cell activation and cytotoxicity.

**Fig. 6:**
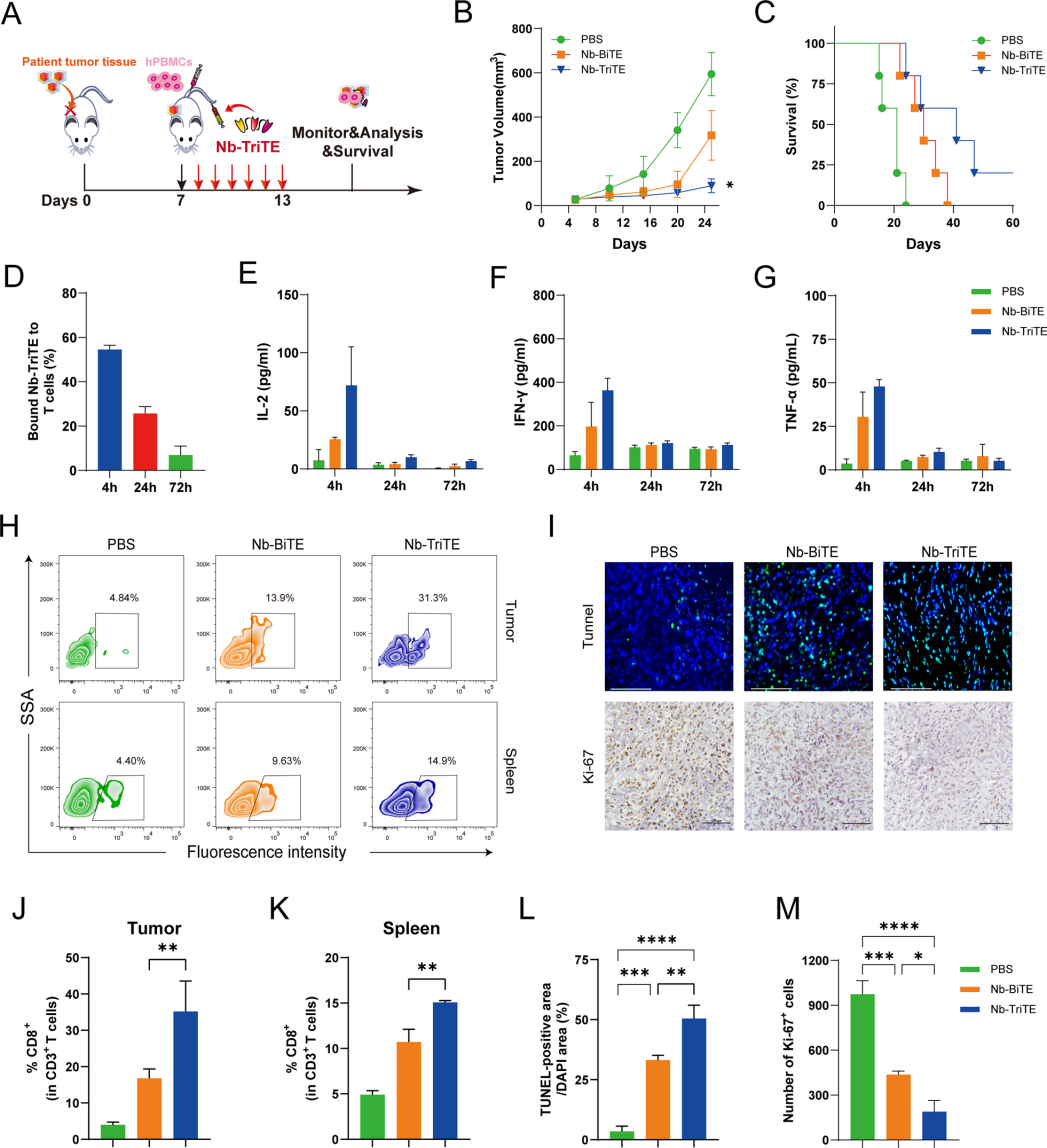
Nb-TriTE treatment effectively inhibits the growth of PDX tumors. **A** Schematic experimental design of the PDX tumor model studies. Tumor growth (**B**), and survival curves (**C**) in PDX models (*n* = 5 per group) treated daily with either Nb-TriTE or Nb-BiTE for 6 consecutive days. **D** The dynamic changes in the proportion of T cells bound to Nb-TriTE within 72 h after treatment. **E**–**G** The levels of IL-2 (**E**), IFN-γ (**F**) and TNF-α (**G**) after treatment with the Nb-TriTE or Nb-BiTE. CD3^+^ CD8^+^ T cell infiltration in the tumor tissues and spleen based on flow cytometry analysis, representative flow cytometry density plots (**H**) and statistical bar chart (**J**, **K**). TUNEL and Ki67 staining of tumor tissues. Representative images (**I**) and statistical bar chart (**L**, **M**). TUNEL staining of tumor tissues. Scale bars, 125 μm; Ki67 IHC of tumor tissues. Scale bars, 100 μm. (*n* = 3 per group)

To better mimic the environment of tumor progression in vivo, a PDX model was established using patient liver cancer tissue (Fig. 6A). FAP and PD-L1 expression in PDX tumors was measured by flow cytometry showed that their high expression (Additional file 1: Figure S7H). In the liver cancer PDX model, we observed an inhibition of tumor growth and longer survival time in mice after daily Nb-TriTE administration compared to their counterparts receiving Nb-BiTE or PBS treatment (Fig. 6B, C). Neither mouse model showed obvious body weight changes before or after the treatment (Additional file 1: Figure S7D). The line graph depicts the tumor volume in the PDX model (Additional file 1: Figure S7G). Analysis of Nb-TriTE binding to the T cells from the animals showed a moderate percentage bound to T cells within 24 h (Fig. 6D). We also found that the dynamic serum levels of IL-2, IFN-γ and TNF-α in the Nb-TriTE-treated NOD/SCID mice showed a transient upregulation (Fig. 6E–G). To further assess the ability of the Nb-TriTE to promote immune cell infiltration. Compared to Nb-BiTE- and PBS-treated mice, Nb-TriTE-treated mice had an increased number of human CD3^+^ CD8^+^ T cells in the tumor and spleen after treatment (Fig. 6 H, J, K). We also analyzed the content of T cells in peripheral blood by flow cytometry. The percentage rate of CD3^+^ T cells was significantly higher in Nb-TriTE-treated mice within 2 weeks after injection (Additional file 1: Figure S7I, J). We then performed TUNEL staining and Ki67 IHC staining of tumor tissues from different groups (Fig. 6I). Interestingly, the Nb-TriTE-treated group exhibited a reduced number of Ki67-positive cells and an increased number of cells undergoing apoptosis according to TUNEL staining compared to those in Nb-BiTE and PBS groups (Fig. 6L, M). Overall, the PDX model experiment showed that the Nb-TriTE could be an effective strategy to treat cancer. Altogether, these results demonstrated that this novel Nb-TriTE format offers a favorable therapeutic platform and exert enhanced therapeutic effects.

### Safety profile of the Nb-TriTE

**Fig. 7:**
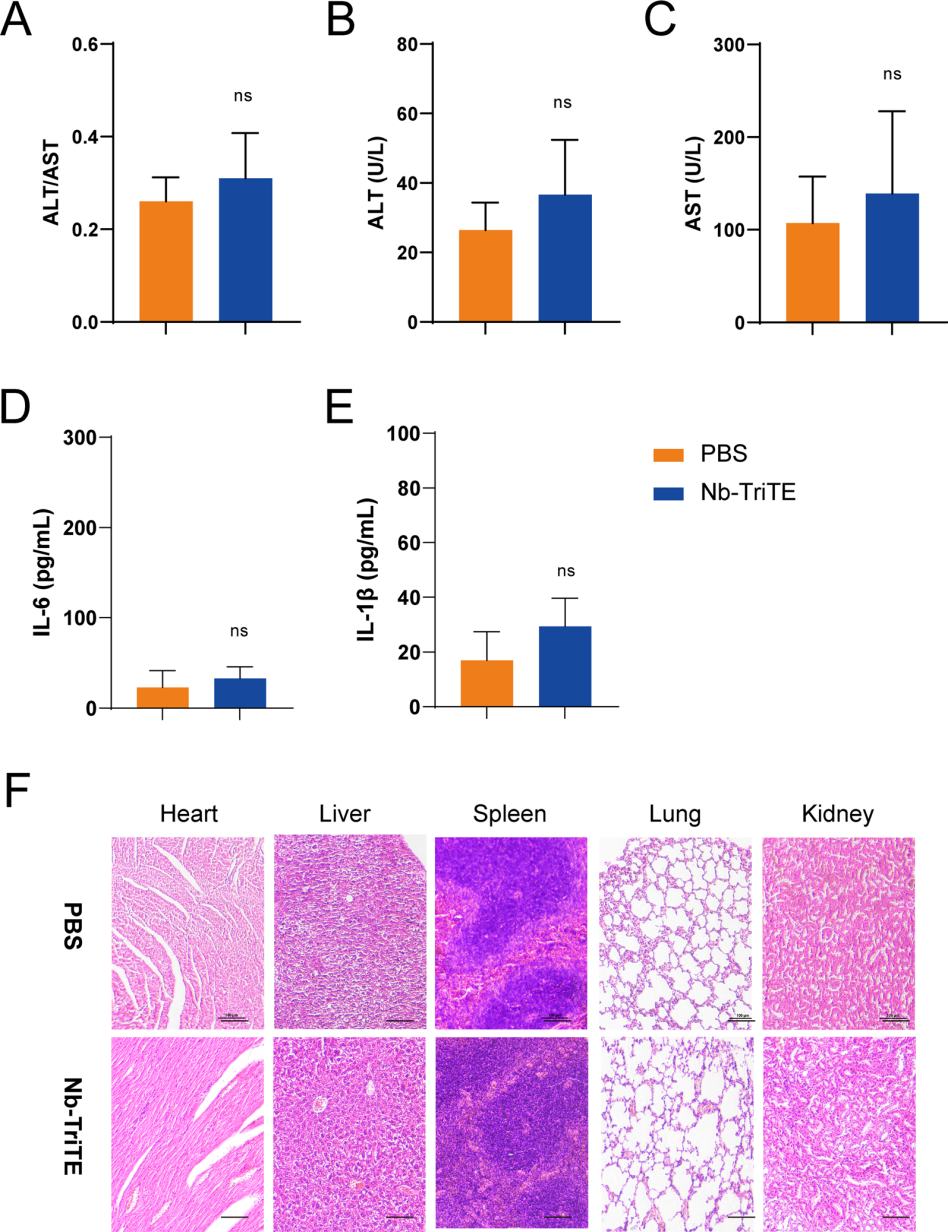
The preliminary safety profile of the Nb-TriTE. ALT/AST ratios (**A**) and ALT (**B**) and AST (**C**) levels in the experiment. The levels of IL-6 (**D**) and IL-1β (**E**) after treatment. **F** Representative HE staining of the major organs (heart, liver, spleen, lung, kidney) of mice after different treatments. Scale bar, 100 μm. No obvious histological difference was observed after Nb-TriTE treatment

We then determined the safety profile of the Nb-TriTE. Despite the absence of any signs of discomfort or toxicity in the mice, we analyzed the major organs (heart, liver, spleen, lung, kidney) for cytopathy by HE staining, and no obvious lesions or organic injuries were present in the Nb-TriTE group compared to the PBS group (Fig. 7F). Moreover, there was no significant increase in the ALT or AST levels or the ratio of ALT/AST in the blood of mice treated with the Nb-TriTE (Fig. 7A–C). In addition, there were no significant differences in the IL-6 (Fig. 7D) and IL-1β (Fig. 7E) levels in the Nb-TriTE group compared with the PBS group, indicating that Nb-TriTE has a manageable safety profile in mice. In summary, these findings demonstrated that the Nb-TriTE is safe to use and support further clinical development.

## Discussion

**Fig. 8:**
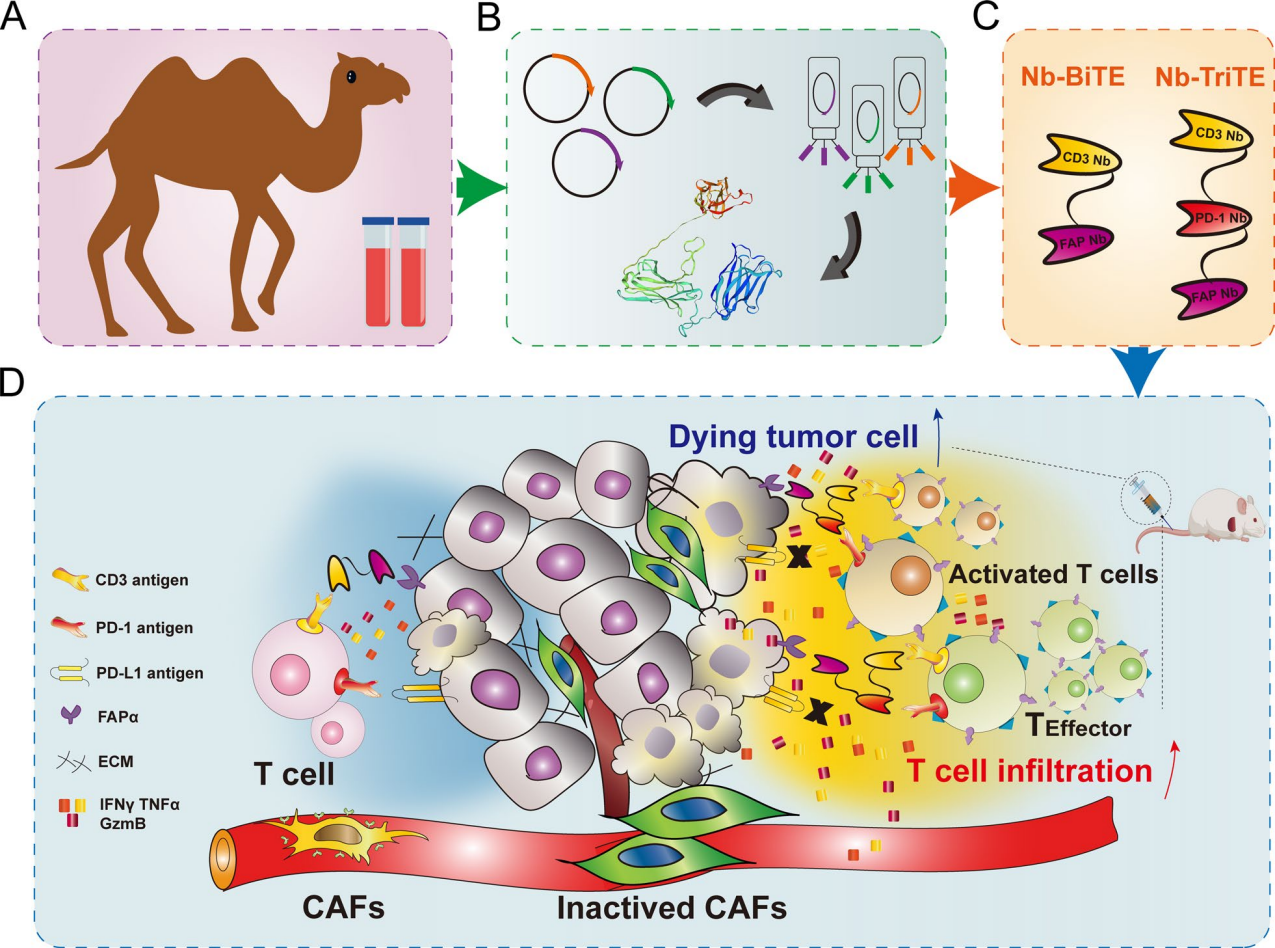
Schematic diagram demonstrating the enhanced antitumor effect of Nb-TriTE. **A**, **B** Preparation of the Nb. **C** Generation and structures of the Nb-BiTE and Nb-TriTE. **D** Working model of the Nb-BiTE and Nb-TriTE

The development of novel TCE formats is imperative given the challenges that in using conventional BiTEs for the clinical treatment of solid tumors. In this study, we innovatively developed Nb-TriTE, a novel therapeutic platform with three antigen binding sites that bind T cells and specific tumor targets together with immune checkpoint targets to potentially recondition the immunosuppressive TME and enable effective T cell antitumor activity (Fig. 8A–D). The Nb-TriTE is the first proposed Nb-only candidate to simultaneously target CD3ε, solid tumor site and immune checkpoint, while this Nb-TriTE format differs from previously reported formats, such as TriTE [47], TriTAC [48, 49], DARPin [34], CiTE [33] and other trispecific antibodies for cancer immunotherapy [46]. These strategies represent a novel and significant advance to current immunotherapy approaches.

T cell-mediated immunity is crucial for solid tumor immunotherapy. Tumor cells escape immune surveillance through molecular mechanisms associated immunosuppression, which attenuates T cell responses and promotes tumor invasion and intravasation [50, 51]. Alternatively, one of the reasons for the ineffectiveness of conventional TCE approaches is mainly due to the unilateral focus on recognizing tumor antigens to kill tumor cells and the lack of consideration for overcoming TME immune suppression and reversing T cell dysfunction. However, interactions between immune checkpoints and their ligands negatively regulate T cell activation pathways, leading to reduced CD8^+^ T cell antitumor immunity via irreversible T cell exhaustion. Recently, it has been shown that solid tumors often escape BiTE-mediated T cell function by upregulating the PD-1/PD-L1 immune checkpoint and subsequently transducing inhibitory signals to T cell [52–54]. Detection of PD-L1 expression in tumor tissues could be explored as a predictive biomarker response to Nb-TriTE in a potential clinical setting. Public data analysis revealed that FAP expression has significant immune correlations with multiple tumor tissues, and single-cell analysis data show the specific expression of FAP in CAFs from PAAD patient (Additional file 1: Figure S11A–D). Indeed, CAF depletion may be enough to exert an indirect therapeutic effect given that FAP is upregulated on CAFs across a broad range of solid malignancies, appealing the idea that Nb-TriTE simultaneously targets cancer cells and immunosuppressive CAFs for T cell killing. It has been suggested that FAP^+^ CAFs are responsible for resistance to anti-PD-1/PD-L1 immunotherapy [55], which supported the rationale behind the combination of anti-FAP and anti-PD-1 VHH in the Nb-TriTE. Therefore, it is crucial to explore the potential of Nb-based agents to enhance T cell function and TCE molecule activity by preventing this immunosuppressive signaling interaction.

Moreover, Nbs hold great promise for clinical applications owing to their good stability and affinity, their ability to be easily expressed and genetically manipulated in various expression systems for large-scale production and their relatively low cost, showing excellent developability. In the current study, we successfully screened a tumor-specific antigen Nb (FAP Nb) with good specificity and affinity for the first time. We verified the high binding affinity of six FAP Nbs by flow cytometry and SPR assays, and selected FAP Nb4 with good affinity and high yield for further investigation. In addition, we obtained several candidate Nbs (PD-1 Nb and CD3ε Nb) in previous research [19, 41]. Successful screening of these Nb candidates lays the foundation for the development of single or multiple Nb-based targeted therapies in future studies. Accordingly, we then selected three specific Nbs to construct the Nb-TriTE platform that specifically targets FAP in CAFs and CD3ε in T cells as well as PD-1 immunomodulatory molecules. Subsequently, we generated a novel Nb-TriTE or Nb-BiTE with suitable antigen specificity and binding ability. The Nb-TriTE is not only more effective in enhancing T cell proliferation, activation and cytotoxicity in vitro than the Nb-BiTE, but also suppress tumorigenesis in multiple mouse models of solid tumors. Interestingly, the Nb-TriTE + hPD-1 was significantly less effective than Nb-TriTE alone in mediating T cell activation, proliferation, and lower secretion of proinflammatory cytokines under antigen stimulation in vitro, which may explain why Nb-TriTE has better performance in targeting immune checkpoint molecules to stimulate antitumor T cell responses. We also observed that the Nb-TriTE induces the specific lysis of FAP^+^ tumor cells in vitro in a dose-specific and antigen-specific manner, whereas FAP^−^ cells are not affected. Indeed, the PD-1 blockade assay highlighted the indispensable role of targeting the PD-1/PD-L1 axis in Nb-TriTE therapy to rejuvenate a sustained T cell immune response against solid tumors. These results indicated that the Nb-TriTE exhibits suitable antigen binding and good affinity, which shows the broad clinical application for Nb-based TCEs, alone or in the form of combination therapies for cancer immunotherapy.

Given the above, we demonstrated that Nb-BiTE treatment had limited antitumor efficacy compared with Nb-TriTE treatment in vivo. Nb-TriTE treatment could generate a more sufficient antitumor effect both in vitro and in vivo. Thus, these results suggest that targeting PD-1 blockade to reactivate T cells can elicit more effective antitumor immunity while providing a more direct bridge between T cells and CAFs. Ultimately, in PDX tumor models, Nb-TriTE treatment outperforms Nb-BiTE treatment with increased antitumor efficacy and more CD3^+^CD8^+^ T cell infiltration in the tumor and spleen, and immune checkpoint blockade not only leads to reversal of T cell inhibition but also affects the other immune cells, such as dendritic cells (DC). In addition, a preliminary assessment of the safety profile has shown that the Nb-TriTE has a favorable safety profile. Importantly, we believe that in addition to Nb-TriTE and Nb-BiTE platforms, Nb-based therapy combination strategies such as CAR -T-cell therapy, oncolytic virus (OV)-armed [56], and vaccines [57] with various immune checkpoint antibodies can maximize the benefits of cancer immunotherapy.

Although the tumor control observed in multiple subcutaneous and orthotopic NOD/SCID models is encouraging, these results also underscore the need for further investigation to advance this approach into the clinic. Future studies are required to extend Nb-TriTE half-life by fusion of an albumin-specific Nb for administration at longer intervals to improve patient convenience. Despite the expected low immunogenicity of Nb-based agents, humanization is routinely performed as part of their development for future clinical translation. Looking forward, Nb-TriTE can also be investigated in an immunocompetent mouse model expressing a humanized CD3ε chain (huCD3ε mice) to reveal the key factors that govern clinical responses. Last, single-cell RNA-seq could be performed to provide even greater insight into the transcriptional changes occurring at a single-cell level after Nb-TriTE treatment. Currently, the precise role of Nb-TriTE involved in the T cell effector pathways remains undefined, and further efforts will also need to be directed at the elucidating molecular mechanisms in vivo.

## Conclusions

In this study, we proposed a novel Nb-TriTE platform that combined TCE therapy with immune checkpoint blockade and effectively alleviated tumor-mediated immunosuppression. We concluded that this novel Nb-TriTE is a safe and effective strategy that establishes a proper antigen-specific T cell response to control tumor progression. This new Nb-TriTE platform is universal, flexible targeting strategy with broad applications and can be easily modified for use in patients with a wide range of tumor types. Thus, the Nb-TriTE represents a novel contribution to cancer TCE immunotherapeutics, with broad reaching implications for both and basic immunology and clinical oncology study.

### Supplementary Information

**Additional file 1**. Nanobody-based trispecific T cell engager (Nb-TriTE) enhances therapeutic efficacy by overcoming tumor-mediated immunosuppression.

## Data Availability

The authors confirm that the data supporting the findings of this study are available within the article (and/or its supplementary materials).

## Abbreviations

TCE: T cell engager
BiTE: Bispecific T cell engager
TriTE: Trispecific T cell engager
Nb: Nanobody
scFv: Single-chain variable antibody fragments
TME: Tumor microenvironment
FAP: Fibroblast activation protein
CAFs: Cancer-associated fibroblasts
PD-1: Programmed cell death 1
PBMCs: Peripheral blood mononuclear cells
NOD/SCID: Non-obese diabetic/severe combined immunodeficiency
IPTG: Isopropyl β-D-1-thiogalactopyranoside
SPR: Surface plasmon resonance
PBS: Phosphate buffered saline
SDS‒PAGE: Sodium dodecyl sulfate polyacrylamide gel electrophoresis
ELISA: Enzyme-linked immunosorbent assay
CFSE: 5,6-Carboxyfluorescein diacetate, succinimidyl ester
PI: Propidium iodide
CDX: Cell-derived xenograft
PDX: Patient-derived xenograft
PCR: Polymerase chain reaction
TUNEL: Terminal deoxynucleotidyl transferase dUTP nick-end labeling
IHC: Immunohistochemistry
HE: Hematoxylin–eosin
ALT: Alanine aminotransferase
AST: Aspartate transaminase
