# Supplementary material for: Nanobody-based trispecific T cell engager (Nb-TriTE) enhances therapeutic efficacy by overcoming tumor-mediated immunosuppression
Source: J Hematol Oncol. 2023 Nov 29;16:115. doi: 10.1186/s13045-023-01507-4 (PMC10688028; doi:10.1186/s13045-023-01507-4)
Supplement: Supplementary file 1 — Additional file 1. Nanobody-based trispecific T cell engager (Nb-TriTE) enhances therapeutic efficacy by overcoming tumor-mediated immunosuppression. [file 13045_2023_1507_MOESM1_ESM.docx]

Additional file 1

**Nanobody-based trispecific T cell engager (Nb-TriTE) enhances therapeutic efficacy by** **overcoming tumor-mediated immunosuppression**

Ziqiang Ding^1#^, Shuyang Sun^1#^, Xuan Wang^2#^, Xiaomei Yang^1^, Wei Shi^1^, Xianing Huang^1^, Shenxia Xie^1^, Fengzhen Mo^1^, Xiaoqiong Hou^1^, Aiqun Liu^1^, Xiaobing Jiang^2^, Zhuoran Tang^1^*, Xiaoling Lu^1^*

^1^ School of Basic Medical Sciences/ College of Stomatology/ Hospital of Stomatology/ Guangxi Key Laboratory of Nanobody Research/ Guangxi Nanobody Engineering Research Center/ Laboratory Animal Center/ Pharmaceutical College/ Affiliated Tumor Hospital, Guangxi Medical University, Nanning 530021, China;

^2^ Department of Neurosurgery, Union Hospital, Tongji Medical College, Huazhong University of Science and Technology, Wuhan 430022, China;

^#^ These authors contributed equally to this work.

* Correspondence to Xiaoling Lu (luxiaoling@gxmu.edu.cn), Zhuoran Tang (soup1990@126.com).

**This file includes Figure S1 to S11**

**Supplementary material**

**
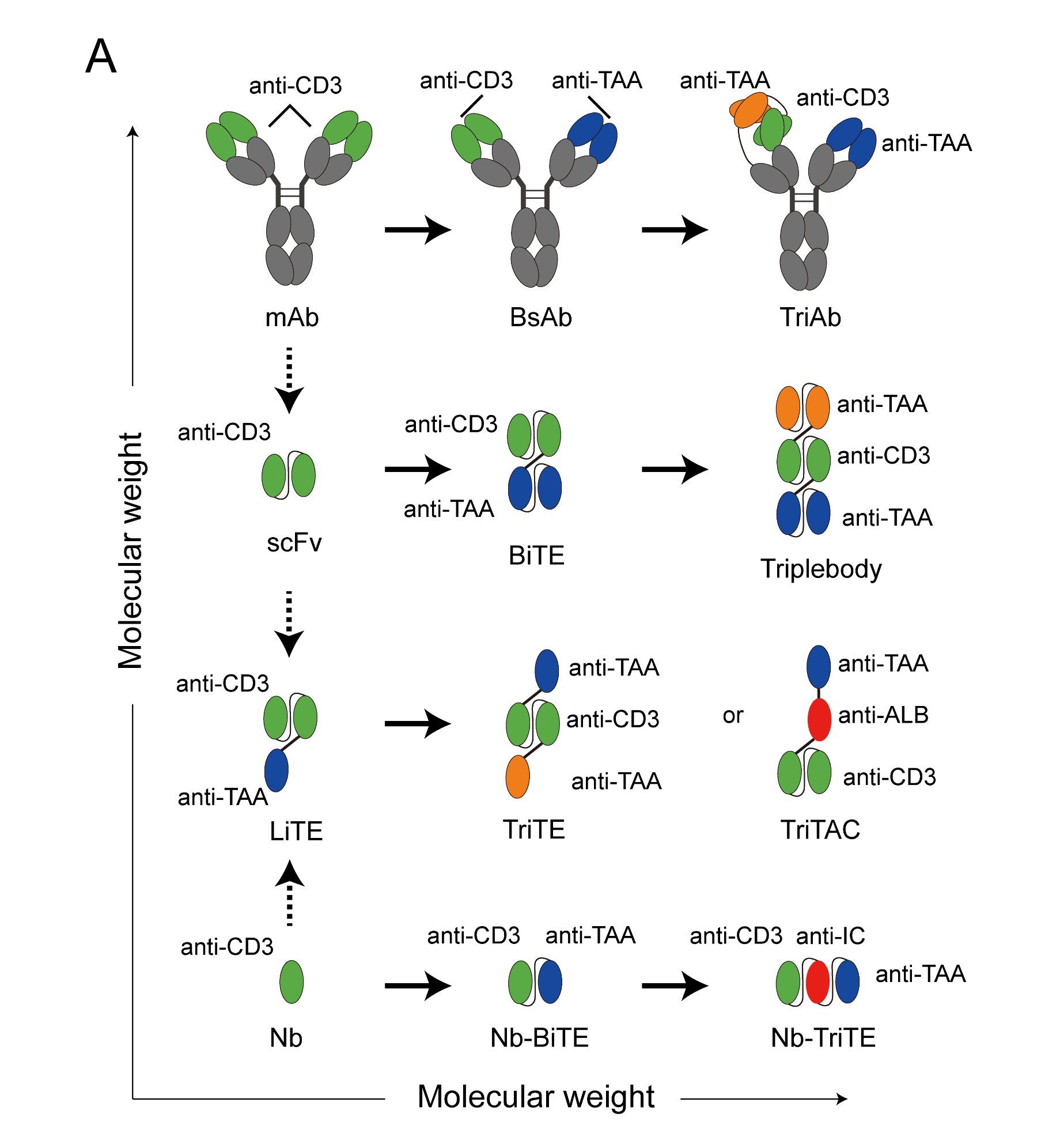
**

**Figure S1. Schematic structures of** **antibodies and their derivates.** (A) Schematic representation of conventional mAb, scFv and Nb and their derivates. Arrows show the evolutionary relationships between indicated antibody types. TAA: tumor associated antigen, IC: immune checkpoint, ALB: albumin, scFv: single chain antibody fragments, Nb: nanobody, BiTE: bispecific T-cell engager, LiTE: light T-cell engager, TriTE: trispecific T-cell engager, TriTAC: trispecific T-cell activating construct, Nb-BiTE: nanobody-based bispecific T-cell engager, Nb-TriTE: nanobody-based trispecific T-cell engager.

**
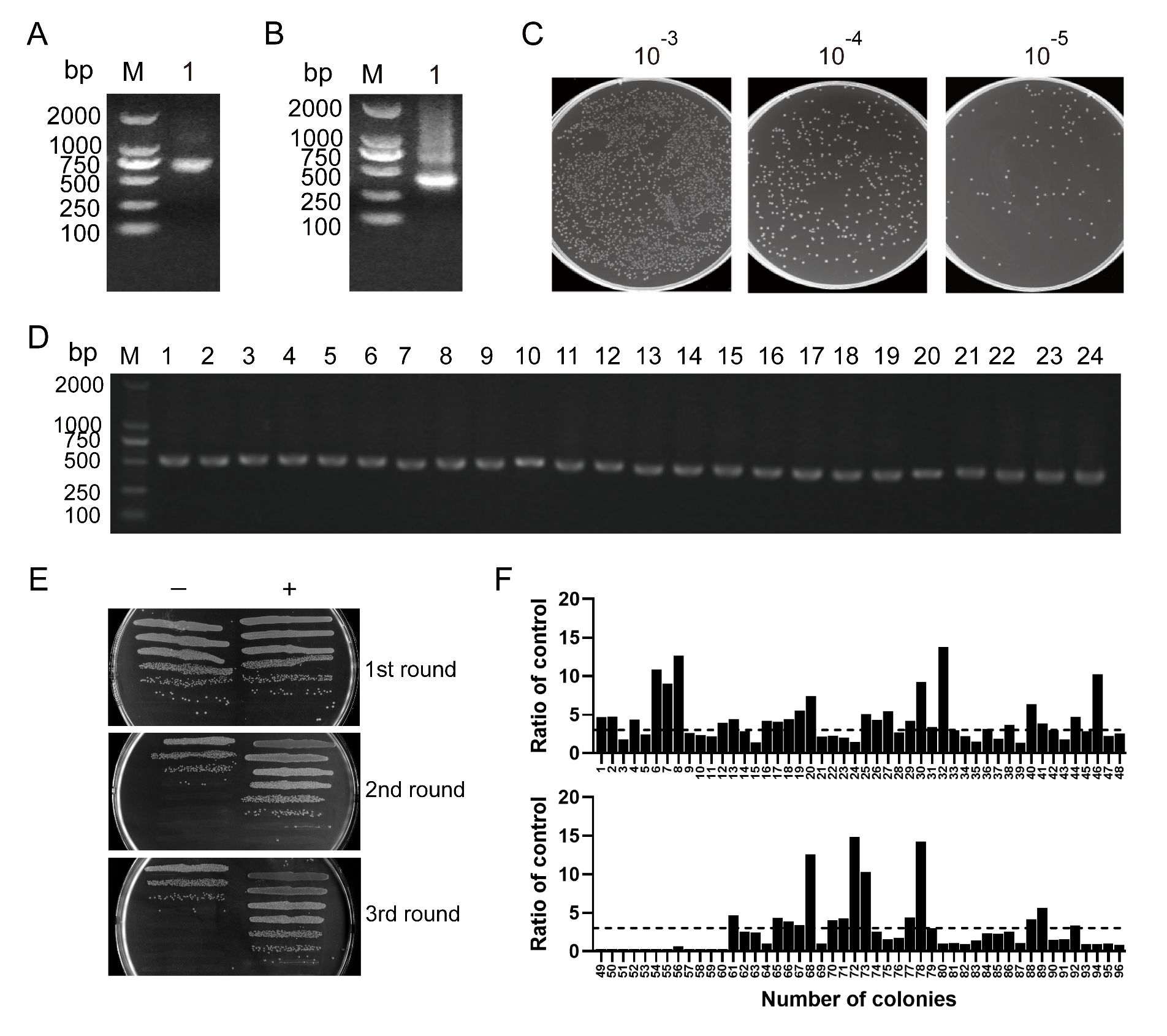
**

**Figure S2. Nb library construction and hFAP Nbs screening.** (A-B) hFAP-specific VHH gene fragments were amplified by a first PCR with a band of ~700 bp (A) and an event band of ~400 bp (B) was amplified by a second, nested PCR. (C) The library capacity was measured by counting the clone numbers after gradient dilution. (D) The correct insertion rate was estimated by 24 randomly selected colonies after PCR amplification. (E) The enrichment of phage particles in the library was determined after three consecutive rounds of panning. (F) Periplasmic extract ELISA was conducted to analyze 96 clones, 24 clones were identified as positive clones (a ratio higher than 3 was considered positive).

**
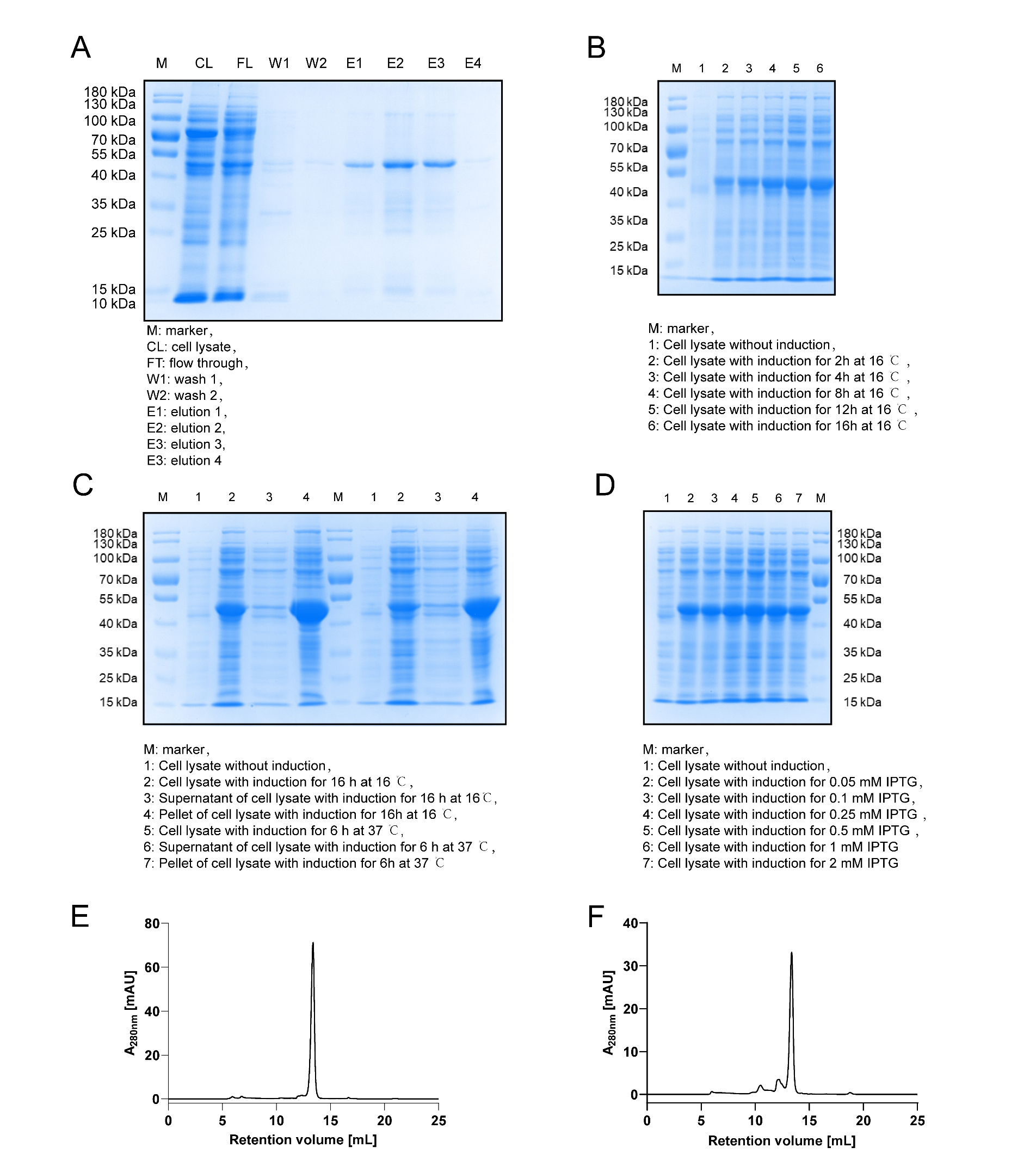
Figure S3. Optimization of induction for maximizing the expressed protein of** **Nb-TriTE.** (A) SDS-PAGE analysis of elution fractions of Nb-TriTE from Nickel column by a stepwise imidazole gradient. Lanes: M- molecular weight marker; CL-cell lysate; FL-flow through; 1- wash 1, 2- wash 2; 3-6-elution 1, elution 2, elution 3, elution 4, respectively. (B-D) Expression of Nb-TriTE under different induced conditions for times and IPTG concentrations. SDS-PAGE analysis identified that the Nb-TriTE was produced in bacteria as an inclusion body after IPTG induction and that the optimum conditions for induction were 0.5 mM IPTG, 16 °C, 16 h. SEC analysis of the purified proteins Nb-BiTE (E) and Nb-TriTE (F).


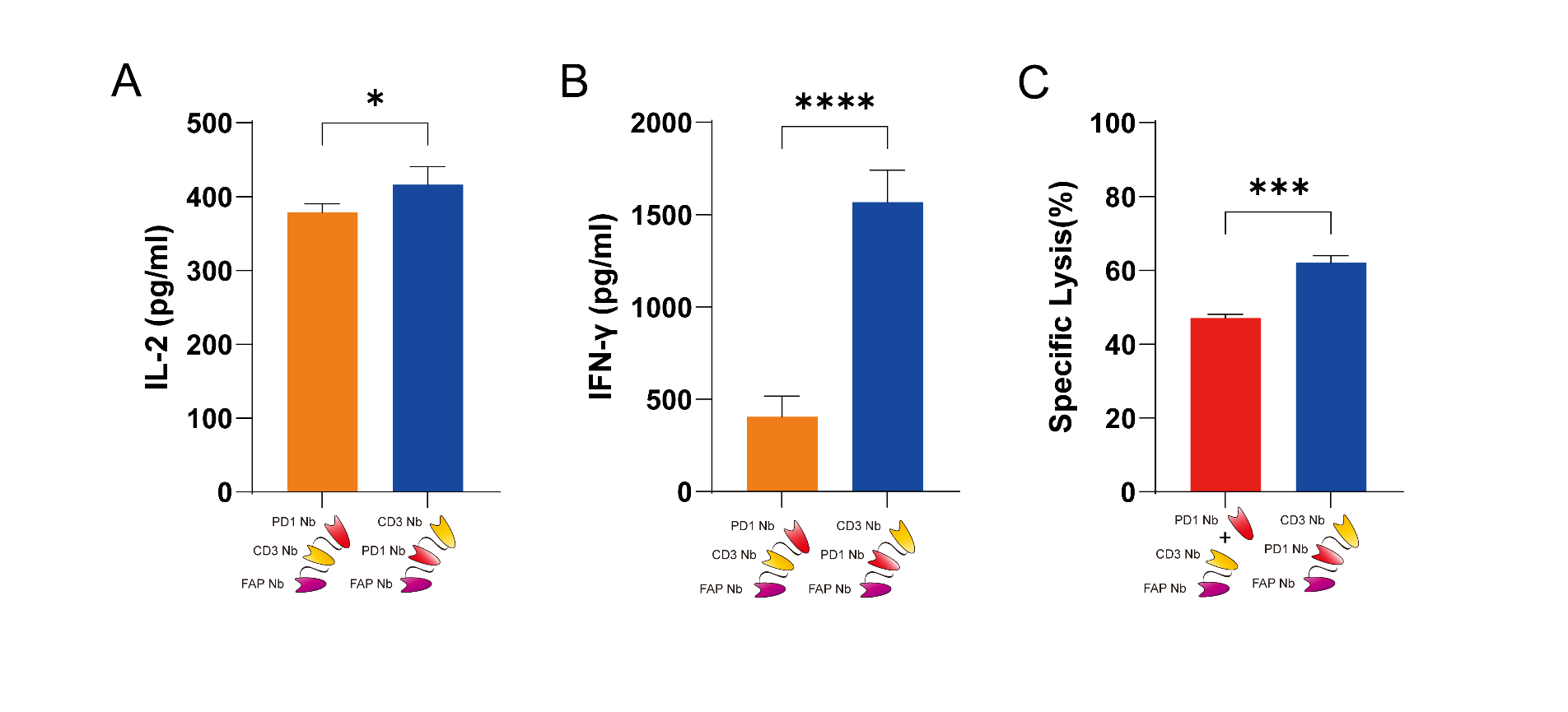


**Figure S4. Optimization of configurations for CD3 Nb and PD-1 Nb in alternative positions of Nb-TriTE.** (A-B) The secretion of by cytokine release assay *in vitro*. IFN-γ and IL-2 in the supernatant were measured by ELISA after coculture with FAP^+^ target HepG2-FAP cells and T cells in the presence of distal-CD3 Nb × proximal-PD-1 Nb or distal-PD-1 Nb × proximal CD3 Nb. (C) The *in vitro* cell lysis assays of Nb-TriTE was measured compared with Nb-BiTE+anti-PD-1 Nb after coculture with FAP^+^ target HepG2-FAP cells and T cells using flow cytometry analysis as above.

**
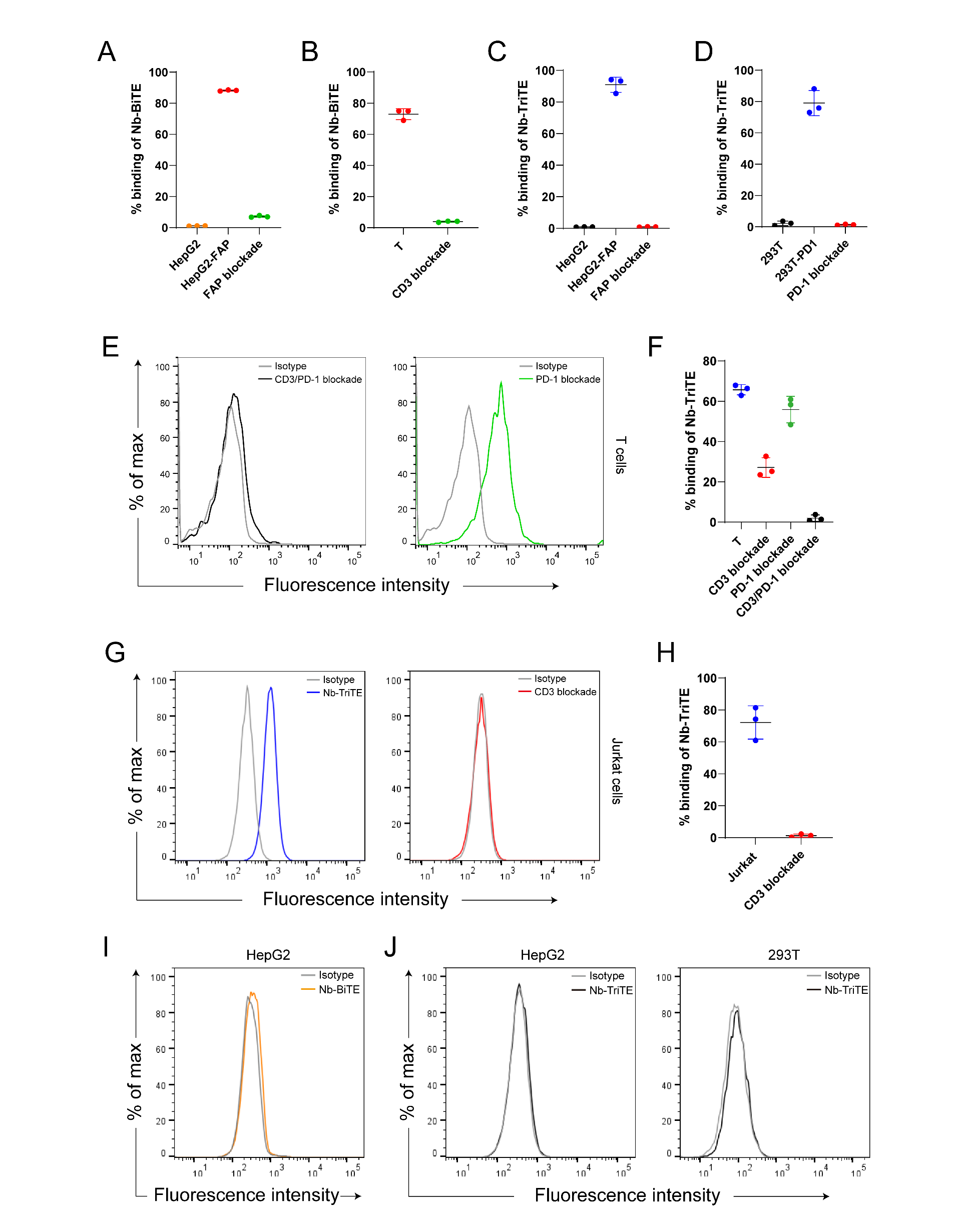
**

**Figure S5. Specific binding ability of Nb-TriTE and Nb-BiTE by flow cytometry.** (A-B) Statistical bar charts of Nb-BiTE binding to HepG2 cells, HepG2-FAP cells and T cells. (C-D) Statistical bar charts of Nb-TriTE binding to HepG2 cells, HepG2-FAP cells, 293T cells and 293T-PD1 cells. (E-F) Representative flow diagrams and statistical bar charts of Nb-TriTE bind to T cells. CD3 blockade, PD-1 blockade and CD3+PD-1 co-blockade as experimental groups. (G-H) Representative flow diagrams and statistical bar charts of Nb-TriTE bind to Jurkat cells. After CD3 blockade, Nb-TriTE could not bind to Jurkat cells. (I-J) Representative flow diagrams of Nb-BiTE and Nb-TriTE binding to FAP^-^ HepG2 cells, Nb-TriTE binding to 293T cells. All data represents mean ± standard deviation from 3 independent experiments.

**
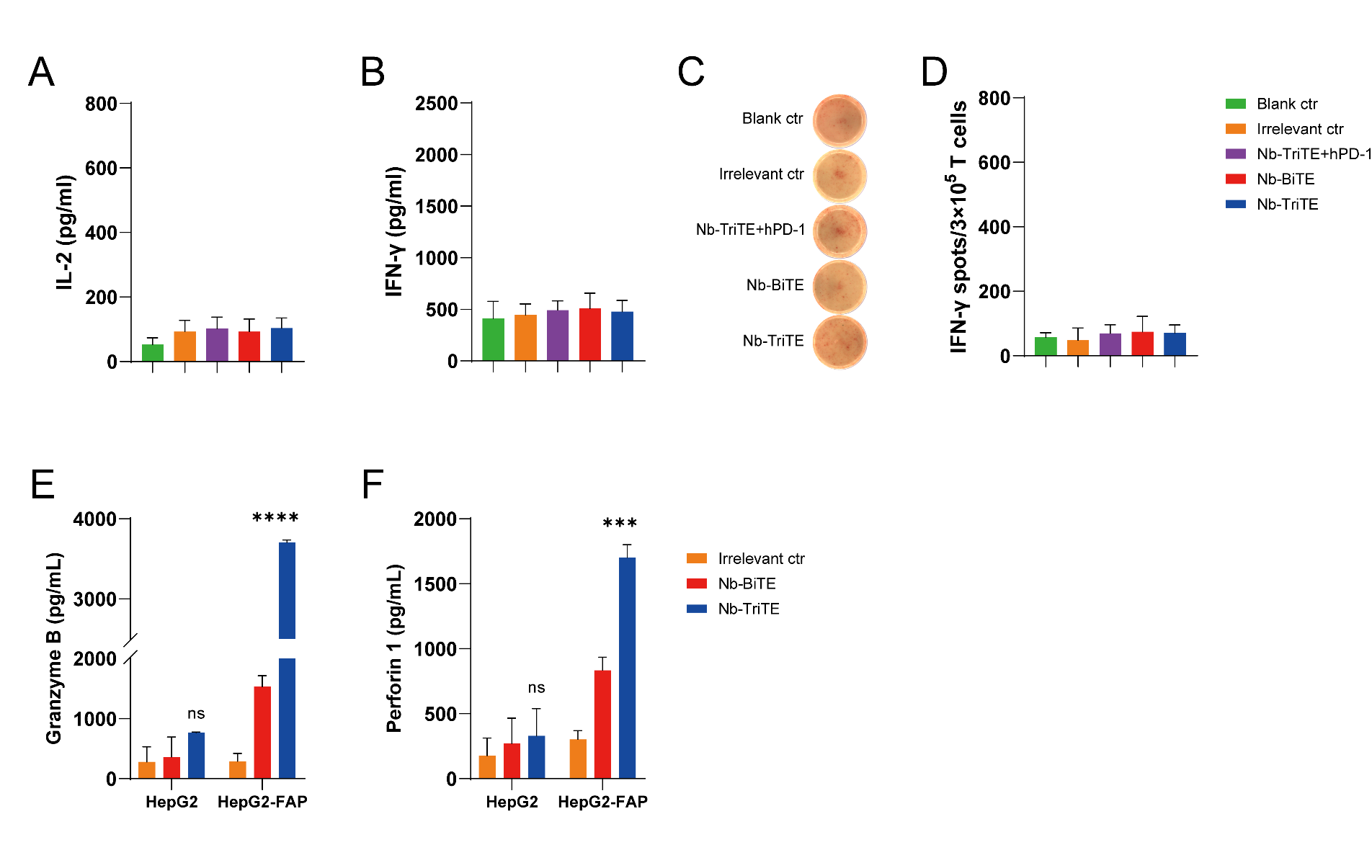
**

**Figure S6. ELISPOT and cytokine secretion assays responses on co-cultured with FAP target cells.** (A-B) Levels of IL-2 and IFN-γ secretion in the presence of FAP^-^ target cell HepG2 upon addition of Nb-TriTE, Nb-BiTE or Irrelevant ctr and Nb-TriTE+hPD-1 at equimolar concentration. (C-D) Representative images of IFN-γ ELISPOT wells from cultured IFN-γ ELISPOT assays. Levels of Granzyme B (E) and Perforin1 (F) secretion by ELISA. All data represents mean ± standard deviation from 3 independent experiments.

**
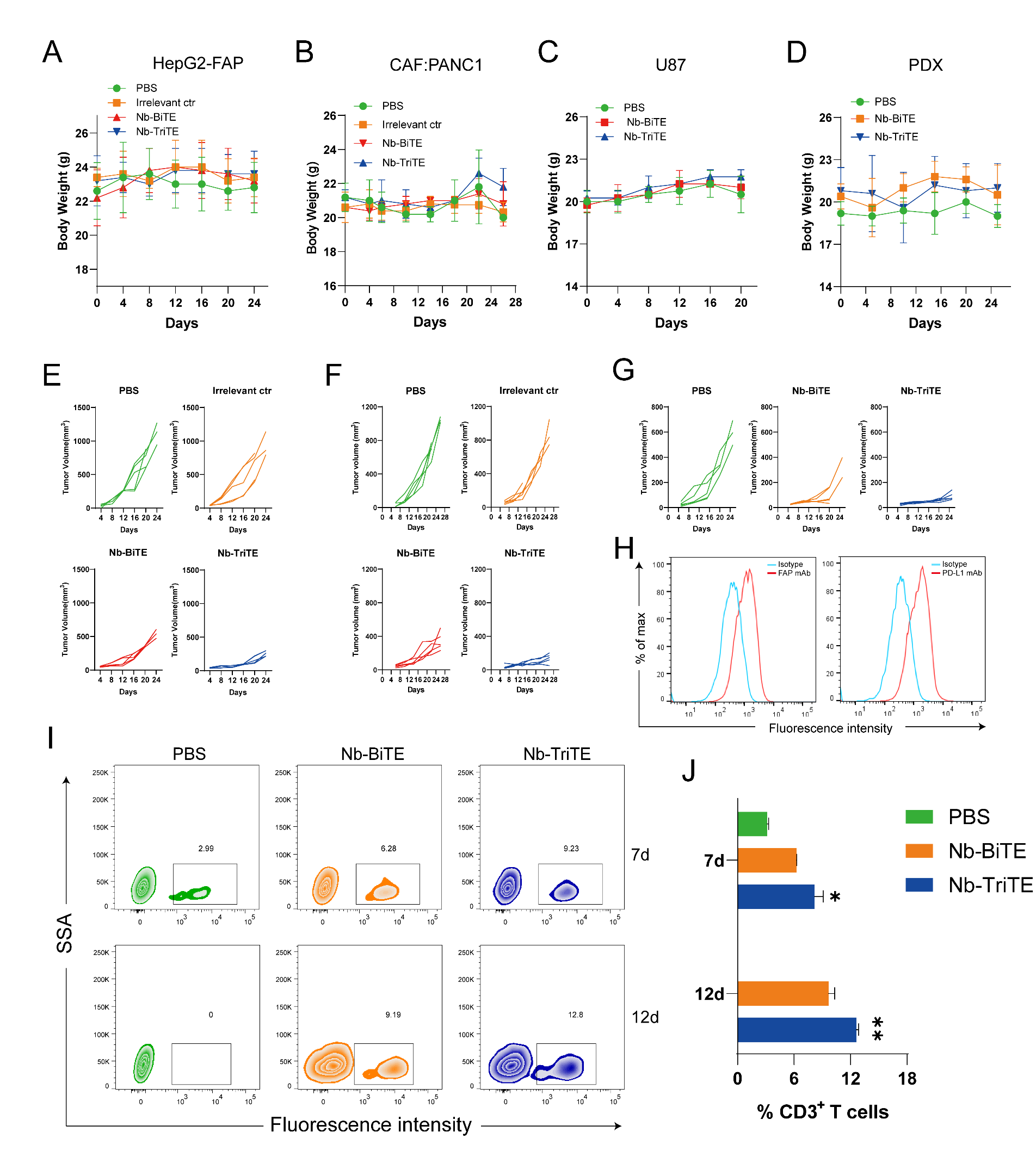
**

**Figure S7. *In vivo* antitumor efficacy of Nb-TriTE in mouse xenograft models.** Body weight of HepG2-FAP (A), CAFs: PANC1 (B), U87 (C), or PDX (D) tumor bearing mice during experiment. Tumor volume of each group for HepG2-FAP (E), CAFs: PANC1 (F) or PDX (G) tumor bearing mice at different times. (H) FAP and PD-L1 expression in PDX tumors was detected by flow cytometry. (I-J) Representative flow cytometry plots and statistical analysis of CD3^+^ T cell content in blood for PDX tumors.

**
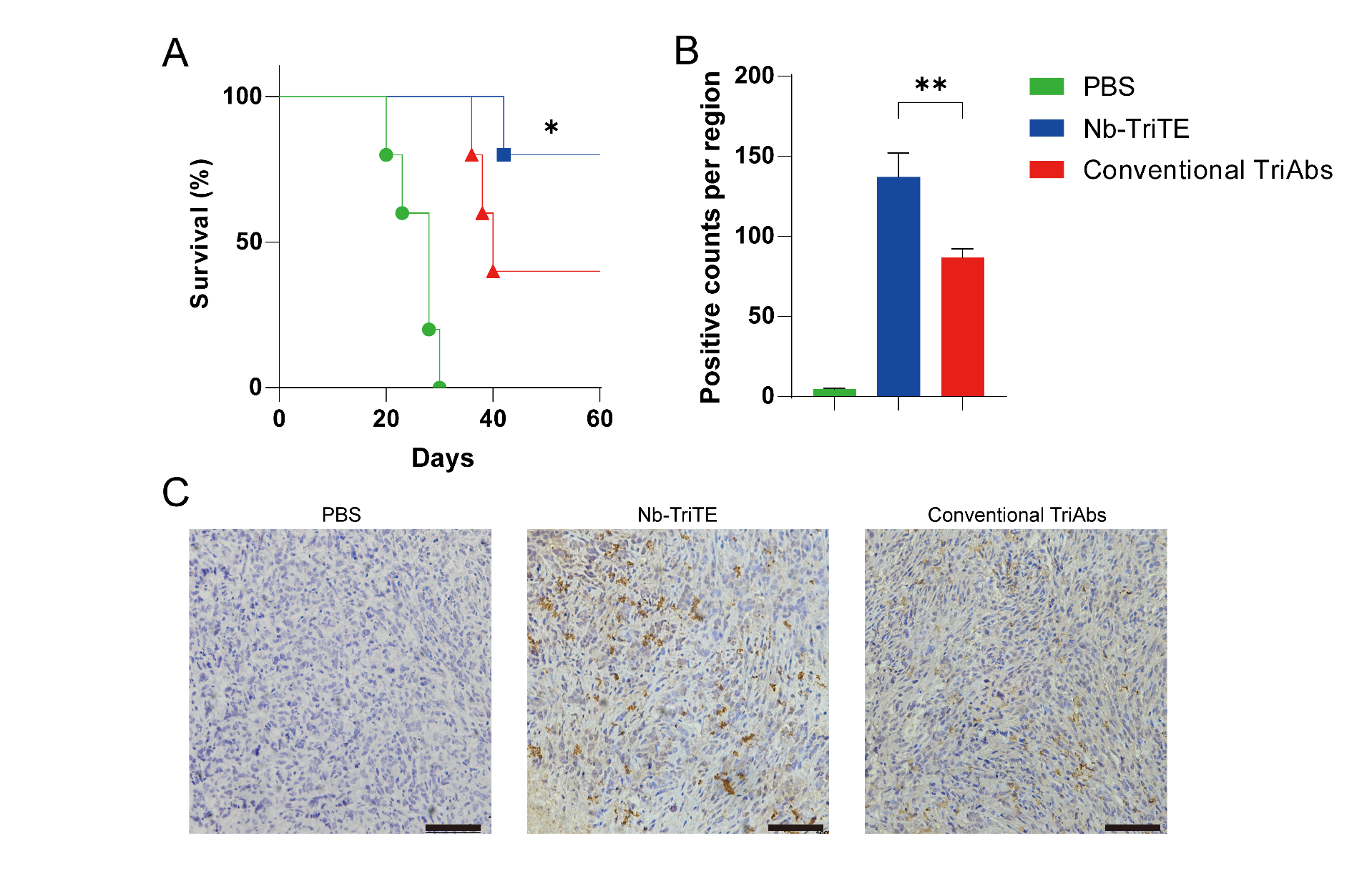
Figure S8. Nb-TriTE has better tissue penetration *in vivo* than conventional trispecific antibodies.** (A) Kaplan‒Meier survival curves (n = 5 per group). (B-C) IHC staining analysis by His-tag antibody in tumor-bearing NOD/SCID mice treated with Nb-TriTE or conventional TriAbs. Scale bars, 100 μm.

**
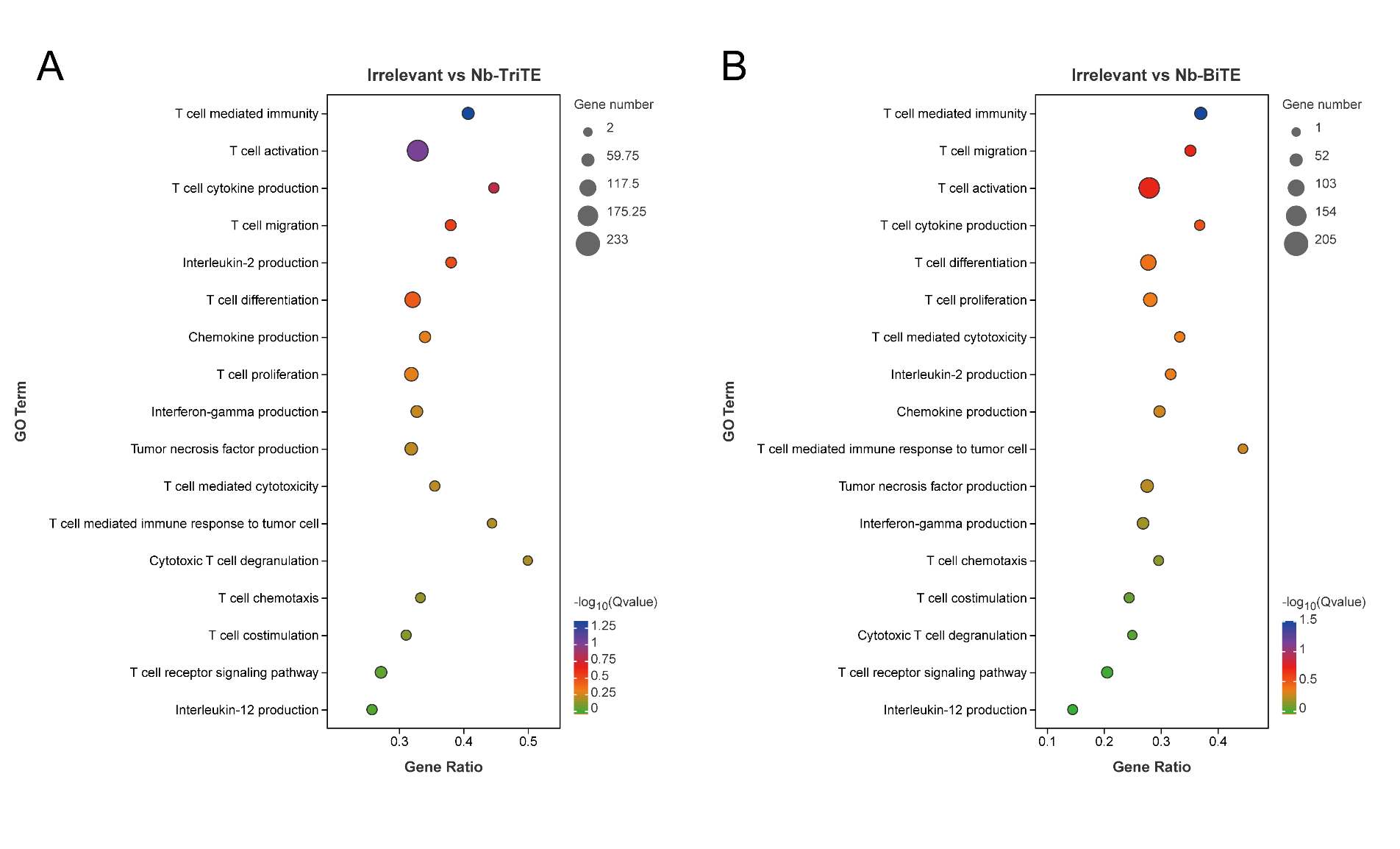
**

**Figure S9. RNA-Seq analysis from mouse CAFs: PANC1 tumor tissues.** (A) GO enrichment analysis between Nb-TriTE and irrelevant group. (B) GO enrichment analysis between Nb-BiTE and irrelevant group.


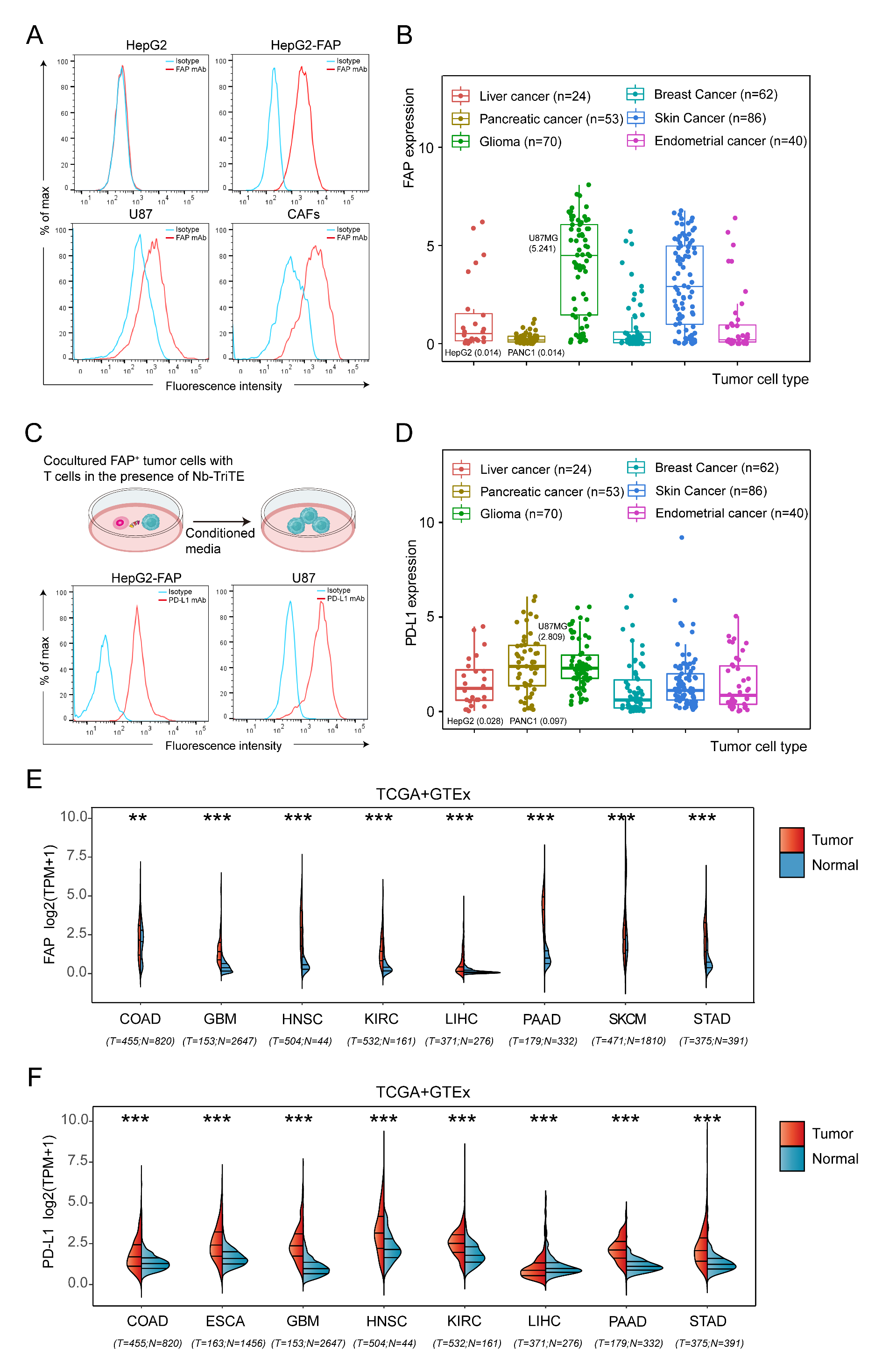


**Figure S10. FAP and PD-L1 expression in target cells and tumor tissues by flow cytometry and publicly datasets.** (A-B) FAP expression in target cells by flow cytometry analysis and CCLE database. (C-D) PD-L1 expression in target cells by flow cytometry analysis and CCLE database. The FAP (E) and PD-L1 (F) expression distribution in tumor tissues and normal tissues from TCGA+GTEx dataset.


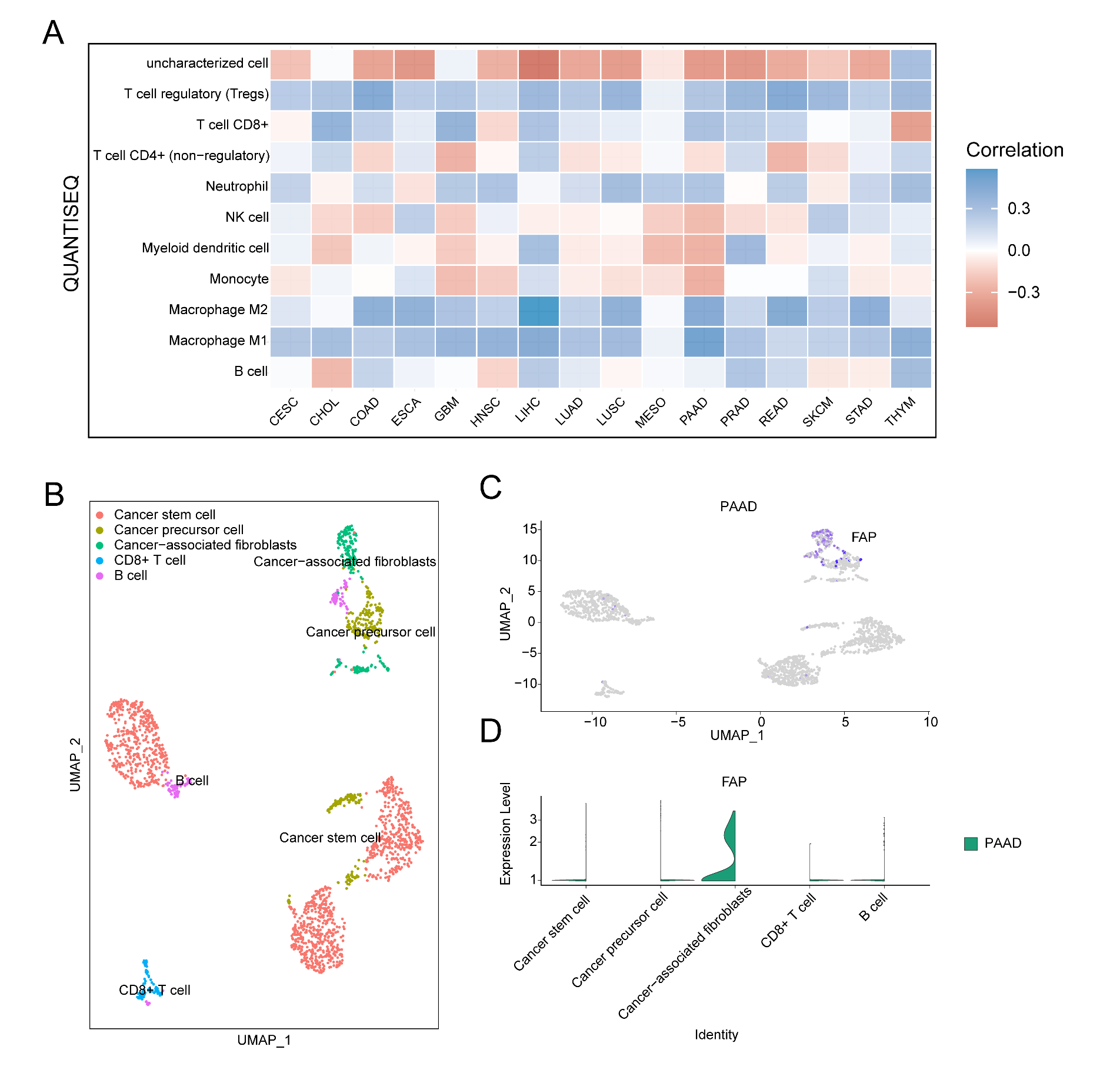


**Figure S11.** **Immune score and single-cell analysis reveal FAP expression in tumor tissues from the public data.** (A) The heatmap of CIBERSORT immune score and FAP gene expression in multiple tumor tissues, such as GBM, LIHC and PAAD have the stronger immune correlations with CD8^+^ T cells. (B-D) Single-cell RNA sequencing data of PAAD from the study GSE154778 in the GEO database reveals the specific expression of FAP in CAFs, UMAP plots of clustering were performed using the R package Seurat and FAP expression level in each cell population.
